# Exploring compositional and predicted functional alterations of gut microbiota in *H. pylori* infection

**DOI:** 10.1038/s41598-025-22788-4

**Published:** 2025-10-29

**Authors:** Noha Salah Soliman, May Sherif Soliman, Heba Sherif Abdel Aziz, Amani Ali El-Kholy

**Affiliations:** Clinical and Chemical Pathology, Faculty of Medicine, Cairo University, Cairo, Egypt

**Keywords:** H. pylori, Gut microbiota, Dysbiosis, Taxonomical composition, Functional pathways, Intestinal disorders, Diseases, Gastroenterology, Microbiology

## Abstract

*H. pylori* infection is prevalent worldwide having detrimental effects on the gastrointestinal tract. *H. pylori* has been linked to compositional variations in gastric microbiota, with insufficient knowledge on extended effect to intestinal microbiota. Moreover, functional potentials of gut microbiota remain underexplored. Aim We sought to investigate the compositional and functional shifts in gut microbiota associated with H. pylori infection. Total 54 stool samples were obtained from H. pylori infected patients tested positive for stool antigen (n = 31) compared to control group (n = 23). All samples were subjected to 16SrRNA next-generation sequencing. By comparison with the control group, the H. pylori-positive group displayed relatively higher mean relative abundance (RA) of phyla of Bacteroidetes (43.59%) and Proteobacteria (8.36%), mainly genera of Bacteroides (22.5%) and Prevotella (13.48%) and species of Bacteroides vulgatus (7%) and E. coli (4%). Other low abundant clinically relevant taxa were more enriched in H. pylori-positive group than controls such as Desulfovibrio, Enterococcaceae, Rikenellacae, and Akkermansia. H. pylori-positive group showed distinctive representaion of Prevotella species (PAC001042_s), Citrobacter and Howardella,, while Blautia genus was exclusively identified in the control group. Both study groups showed comparable microbiota diversities with no significant difference. H. pylori-positive group revealed higher abundance of MAPK signaling functional pathways (FDR = 0.046), along with Cell cycle and Ariginine and proloine pathways (FDR = 0.05) compared to the controls. Our findings inferred compositional and functional alterations in H. pylori-associated gut microbiota that may play role in gut inflammation and malignant transformation observed in H. pylori infection.

**Supplementary Information:**

The online version contains supplementary material available at 10.1038/s41598-025-22788-4.

## Introduction

Infection with *H. pylori* is a highly prevalent condition worldwide, especially in developing countries^1^. *H. pylori* is among the most prevalent bacteria, colonizing and infecting the stomach mucosa of 58% of the global population^2^ with the highest prevalence observed in Africa, potentially exceeding 90% in low-income countries^3^. The overall incidence of *H. pylori* infection in the Middle East countries is intriguing, with prevalence rates ranging from 70% to 90%^4^. Infection rates are substantially influenced by geographical location, attributable to regional variances in sanitation, urbanization, dietary habits and socioeconomic status^5^. *H. pylori* has a profound impact on the gastrointestinal mucosa leading to disorders such as chronic gastritis and peptic ulcer disease^2^. Furthermore, *H. pylori* is recognized as an elevated risk factor for gastric carcinoma^5^, and has been classified as a Class I carcinogen by the World Health Organization (WHO)^6^.

The human gut microbiota is an intricate and dynamic microbial community, often regarded as an independently functioning organ that mediates a physiological symbiotic interaction with its host^7^. The gut microbiome typically has a distinct, well-balanced site-specific composition. A disruption in the natural ecosystem of the human microbiome results in a condition referred to as “dysbiosis”^8^. Recent studies indicate that *H. pylori* infection not only disrupts the balance of microbiota in the gastric mucosa, but also induces distant microbial alterations in the human intestine, thereby emphasizing the significance of studying gut microbiota^9,10^. The disturbance of gut microbiota throughout *H. pylori* infection may be a contributing factor to the onset and exacerbation of various intestinal and metabolic disorders^10^. It is speculated that *H. pylori*-associated changes of the intestinal microbiota may play a role in developing intestinal inflammations, elevating the risk of colorectal cancer in infected patients^5,11^. Moreover, recent studies highlighted that the development of inflammatory bowel disease (IBD) through *H. pylori*- associated modulation of immune responses. Additionally, several extra-gastrointestinal disorders have been linked to *H. pylori* infection, such as iron defciency anemia, type 1 diabetes mellitus, cardiovascular diseases, neurological disorders and vitamin B12 deficiency^2,12^. *H. pylori*-associated intestinal and extra-intestinal illnesses have been postulated to be pertained to *H. pylori*- associated disturbance in the homeostasis of gut microbiota^1^. This relationship involves an intricate mutual crosstalk between *H. pylori* and gut microbiome, encompassing aspects such as *H. pylori* virulence, interactions with immune system, modulations of T- cell responses and metabolic alterations. This ultimately triggers a subsequent chronic systemic inflammatory response^5,13^. Nonetheless, there is still a paucity of human studies, which delve into the functional implications beyond the gut microbiome structure, particularly by analyzing the functional pathways linked *H. pylori* infection^5^. The recent advancement of metagenomics next-generation sequencing (NGS) technology has revolutionized the investigation of human microbiota, although developing nations continue to face constrained resources^8^. Promoting comprehensive studies that elucidate population- specific gut microbiome signatures and functional alterations in *H. pylori* infection can enhance understanding in this area and unveil new avenues for clinical management of infection and its related complications^14^.

Through a comprehensive review of existing literature and published data, we identified a notable scarcity of reports from the Middle East region, including our country, regarding the association between *H. pylori* infection and remote gut microbiota alterations^3^. In this context, the majority of previously published literature in the Middle East consists of review articles that reference studies conducted in other geographical regions, with limited availability of specific national and regional applied clinical researches conducted within these countries^12,13^. Furthermore, none of the fore-mentioned published studies has examined the functional microbiome alterations induced by *H. pylori*, particularly within the gut. While numerous studies have primarily focused on examining the compositional variations of microbial communities, a knowledge gap exists concerning the insights into the functional pathways of gut microbiome promoted by *H. pylori*^1^. In this view, the study aimed to employ metagenomics (NGS) to characterize the taxonomical structure, diversities, as well as to investigate the relevant functional pathways of *H. pylori*-associated gut microbiota compared to healthy controls, seeking to provide a novel contribution from our Middle Eastern country.

## Materials and methods

### Study samples

The present work was conducted on stool samples collected from two study groups: *H. pylori*-positive (cases) and *H. pylori*-negative (controls). The Cases group included patients suffering gastrointestinal manifestations, and who were first diagnosed with *H. pylori* infection by having positive stool detection of *H. pylori* antigen. The Control group comprised healthy undergoing stool analysis for routine check-ups, presenting no gastrointestinal complaints, and testing negative for *H. pylori* antigen. Our study excluded subjects based on the following criteria: history of gastric ulcer or gastrointestinal surgery, prior diagnosis or treatment of *H. pylori* infection, recent use of antacids or proton pump inhibitors (PPI) within the last six months. All Stool samples were collected in sterile containers with no preservatives and directly transported to the laboratory where fresh aliquots were prepared and stored at −80 C° for subsequent molecular analyses using 16SmNGS.

### Ethical statement

This study received ethical approval from the Research Ethics Committee (REC) of the Faculty of Medicine, Cairo University with approval number: N-405–2024. The study was conducted in accordance with the Declaration of Helsinki and all participants provided informed consent.

### Bacterial DNA extraction, metagenomics sequencing and data processing

Bacterial DNA was extracted using the QIAamp DNA Stool Mini Kit (Qiagen, USA) according to the manufacturer’s instructions. Briefly, the process of fecal DNA extraction included the following steps: sample homogenization with ASL buffer, chemical cell lysis, non-DNA substances removal using InhibitEX Tablet, and column DNA purification. Gut microbiome detection was done according to Illumina 16 S metagenomic protocol. Briefly, a first stage PCR targeting the 16 S V3 and V4 regions was done using the KAPA HiFi HotStart Ready Mix (Kapa Biosystems, Boston, MA, USA), with 35 amplification cycles at 60 °C annealing temperature. Amplicons were purified using the Agencourt AMPure XP Kit (Beckman Coulter, Tokyo, Japan) and then tagged in a second stage PCR using the Nextera XT Index indexes Kit (Illumina, San Diego, CA, USA)^15^. Libraries were normalized to 4nM, mixed with 5% PhiX Control Kit v3 (Illumina), and denatured according to the manufacturer’s protocol. MiSeq sequencing (paired-end, 300 bp) was performed using MiSeq Reagent Kit v3 MS-102–3003 (Illumina) (600-cycle format; Illumina)^16^. A negative control (Rnase free water) was included from the extraction step to ensure contamination free workflow.

To further classify, cluster and annotate operational taxonomic units (OUT), fastaQ files were uploaded on Ezbiocloud software program (EzBiome, Inc, USA) (https://help.ezbiocloud.net/ubcg-gene-set/). The raw data obtained from 16SrRNA gene sequencing underwent quality filtering, subsequently followed by Rarefaction analysis to assess sequencing depth. Further analysis was conducted, encompassing taxonomic classification, clustering, and the annotation of operational taxonomic units. The EzBiocloud pipeline for microbiome taxonomic profiling comprises pre-processing step followed by taxonomic profiling process. The pre-processing phase involves merging paired end reads, trimming primers and filtration of low quality reads. The taxonomic profiling process includes extraction of non- redundant reads, identification using EzBiocloud database, chimera detection, OTU picking with 97% cut-off and the subsequent calculation of alpha diversity indices and rarefaction curves^17^.

### Gut microbiota taxonomical and functional analysis

The compositional and functional analyses of gut microbiota were conducted and compared between the two study groups (*H. pylori*-positive and –negative groups), in terms of taxonomical classification, microbial diversities, biomarkers and functional pathways, utilizing the EzBiocloud software database (EzBiome, Inc, USA). The EzBiocloud database is integrated with Illumina’s BaseSpace Sequence Hub, as an application for 16 S-based MTP (microbiota taxonomic profiling) analysis. Microbial composition was classified at phylum, genus and species levels, and was represented as average relative abundance (RA). Microbial diversity was assessed in each group by employing the Shannon index for alpha diversity and the Bray-Curtis dissimilarity index for beta diversity, accompanied by two-dimensional principal coordinate analysis (PcoA) visualizations. In the context of taxonomic biomarker discovery, linear discriminant analysis effect size (LEfSe) was computed and illustrated in a cladogram to highlight the taxa exhibiting the greatest discrimination between the groups under comparison^2^.

The functional analysis of gut microbiota was conducted using the phylogenetic investigation of communities by reconstruction of unobserved states (PICRUSt) to predict the metabolic potentials of microbial communities. Functional pathways and orthologues were inferred based on the resemblance between metagenomic sequences with those in the Kyoto Encyclopedia of Genes and Genomes (KEGG) core databases. This included the KEGG Orthology (KO) database, which identifies molecular functions, and the KEGG PATHWAY database, which employs a three-level hierarchical classification system for functional pathways (levels 1, 2, and 3)^18^.

### Statistical analysis

Statistical analyses were performed utilizing the EzBiocloud software (EzBiome, Inc, USA), which employed Wilcoxon rank-sum test to determine significant differences in microbial abundance and alpha diversity between group comparisons, while PERMANOVA (per-mutational multivariate analysis of variance) was used to evaluate the differences in beta diversity measures among the study groups. A significant p-value was considered at < 0.05. The False Dicovery Rate (FDR) was computed to adjust significant p-values across comparison groups for conservative interpretation, using the Benjamini-Hochberg method, with FDR values < 0.05 deemed significant^19^.

## Results

The 16SmNGS was performed on total 54 stool samples categorized into *H. pylori*-positive (*n* = 31) and *H. pylori*-negative (*n* = 23) groups based on the stool antigen detection. Valid sequencing reads of operational taxonomic units (OTUs) were attained at total of 154,9923 and average 30,633 ±10,874.106. Rarefaction analysis confirmed significant microbial community representation, with an estimated library sequence coverage ranging from 99.32% to 99.97% for valid reads, reflecting accurate representation of the gut microbiota in stool samples. (Supplementary Table 1, Supplementary Fig. 1)

### Composition of gut microbial taxa

**Fig. 1:**
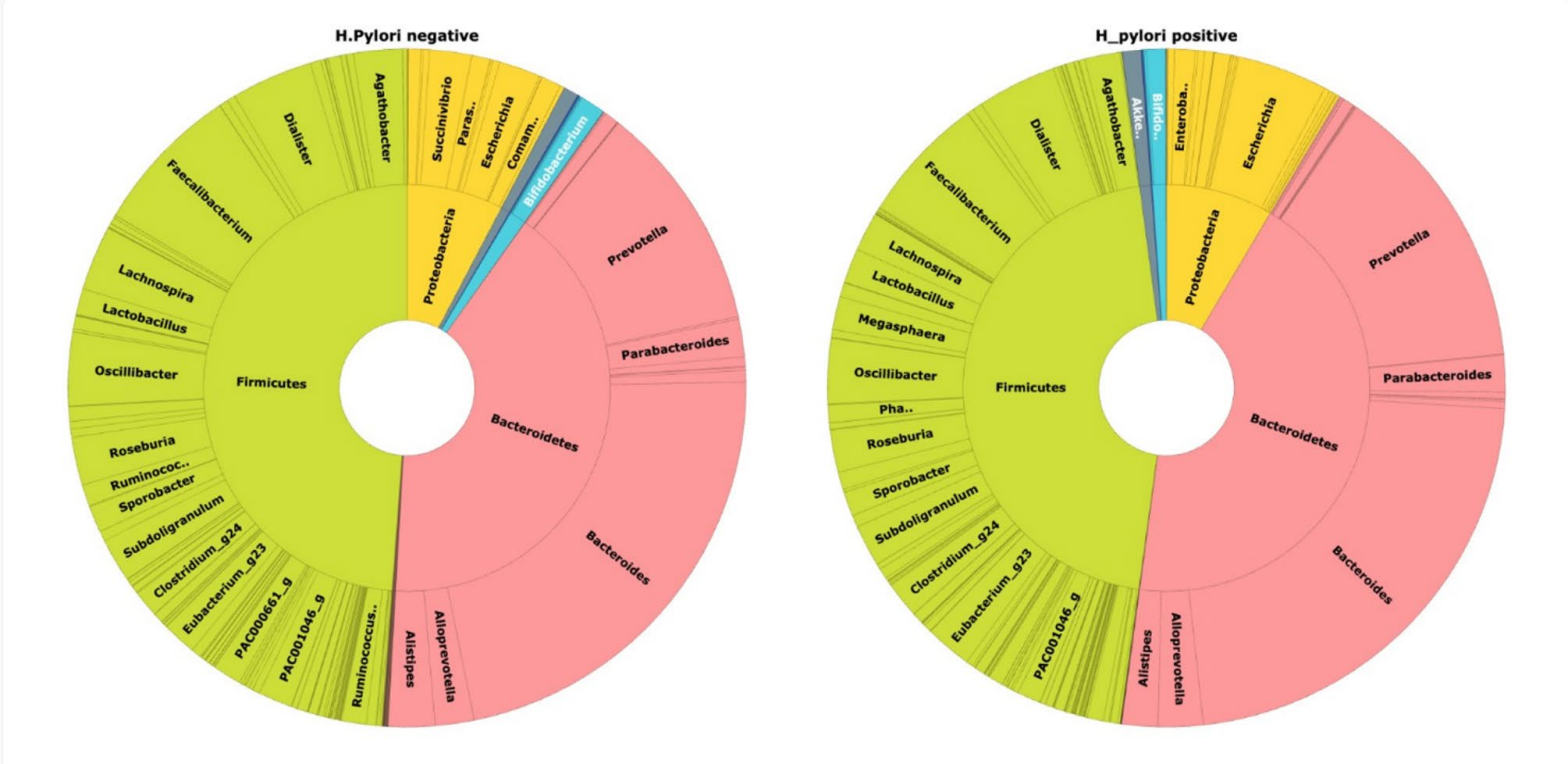
Gut microbiota taxonomic composition among *H. pylori*-positive and -negative groups: Double pie chart showing taxonomic distribution by average relative abundance of gut microbial taxa at the phylum, genus levels between both study groups.

All main dominant taxa in *H. pylori*-positive and *H. pylori*-negative groups were defined by a mean relative abundance (RA) > 1% (Supplementary Table 2). The most prevalent phyla across all sequenced samples, in descending order, were Firmicutes, Bacteroidetes, Proteobacteria, and Actinobacteria. Bacteroidetes and Proteobacteria were more abundant in *H. pylori*-positive (43.59%, 95%CI: 38.11–49.02 and 8.36%, 95%CI: 2.54–14.57, respectively) compared to *H. pylori*-negative groups (41.02%, 95%CI: 35.71–46.32) and 7.69%, 95% CI: 2.73–12.65, respectively), whereas Firmicutes and Actinobacteria showed the opposite trend (Fig. 1 and Supplementary Fig. 2a). Detailed average relative abundances and statistically significant differences among major microbial taxa are provided in Supplementary Table 2. The *H. pylori*-negative group exhibited a higher Firmicutes/Bacteroidetes (F/B) ratio than the *H. pylori*-positive group, though this difference was not statistically significant. Despite its low mean RA (< 1%), the phylum Verrucomicrobia was more abundant in the *H. pylori*-positive group than in the *H. pylori*-negative group (Supplementary Table 3).

Overall, the three most abundant genera in both groups were *Bacteroides*, *Prevotella*, and *Faecalibacterium*. In the *H. pylori*-positive group, *Bacteroides* and *Prevotella* exhibited slightly higher average RA (22.55%, 95%CI: 14.80–29.74.80.74 and 13.48%, 95%CI: 6.69–21.15, respectively), compared to the *H. pylori*-negative group (22.10%, 95%CI: 14.48–29.70 and 10.90%, 95%CI: 4.39–17.41, respectively). Additionally, *Escherichia* was found at higher RA in *H. pylori*-positive group compared to *H. pylori*-negative group. In contrast, *Faecalibacterium* and *Dialister* were relatively more abundant in the *H. pylori*-negative group (Fig. 1, Supplementary Tables 2 and Supplementary Fig. 2b). Other genera exhibited marginal enrichment in either group: *Clostridium_g24*, *Lactobacillus*, *Eubacteria*, *Alloprevotella* and *Megasphaera* were more abundant in the *H. pylori*-positive group, while *Lachnospira*,* Alistipes* and *Bifidobacterium* were more prevalent in the *H. pylori*-negative group (Supplementary Table 2).

At the species level, *Faecalibacterium prausnitzii*, *Prevotella (PAC001304_s)*, *Bacteroides dorei* and *Bacteroides uniformis* were the predominant taxa in the *H. pylori*-negative group, exhibiting higher relative abundance compared to the *H. pylori*-positive group. In contrast, the RA of *Bacteroides vulgatus* (6.66%, 95%CI: 6.65–6.68) surpassed that of *Faecalibacterium prausnitzii* (5.5%, 95%CI: 5.42–5.43) and *Prevotella* (*PAC001304_s)* (5.5%, 95%CI: 5.15–5.53) as the predominant species in the *H. pylori*-positive group. Additionally, *Escherichia coli* and *Dialister* (*PAC001039_s*) exhibited higher average RA in *H. pylori*-positive than *H. pylori*-negative group (Fig. 1, Supplementary Tables 2 and Supplementary Fig. 2c).

Some clinically relevant taxa in literature, although had RA < 1%, still exhibited differential representation in both groups. For example, *Desulfovibrio*, *Enterococcaceae*, *Akkermansia* and *Rikenellaceae* were more abundant in the *H. pylori*-positive group compared to the *H. pylori*-negative group (Supplementary Table 4).

### Taxonomic biomarker discovery and LEfSe analysis

**Fig. 2:**
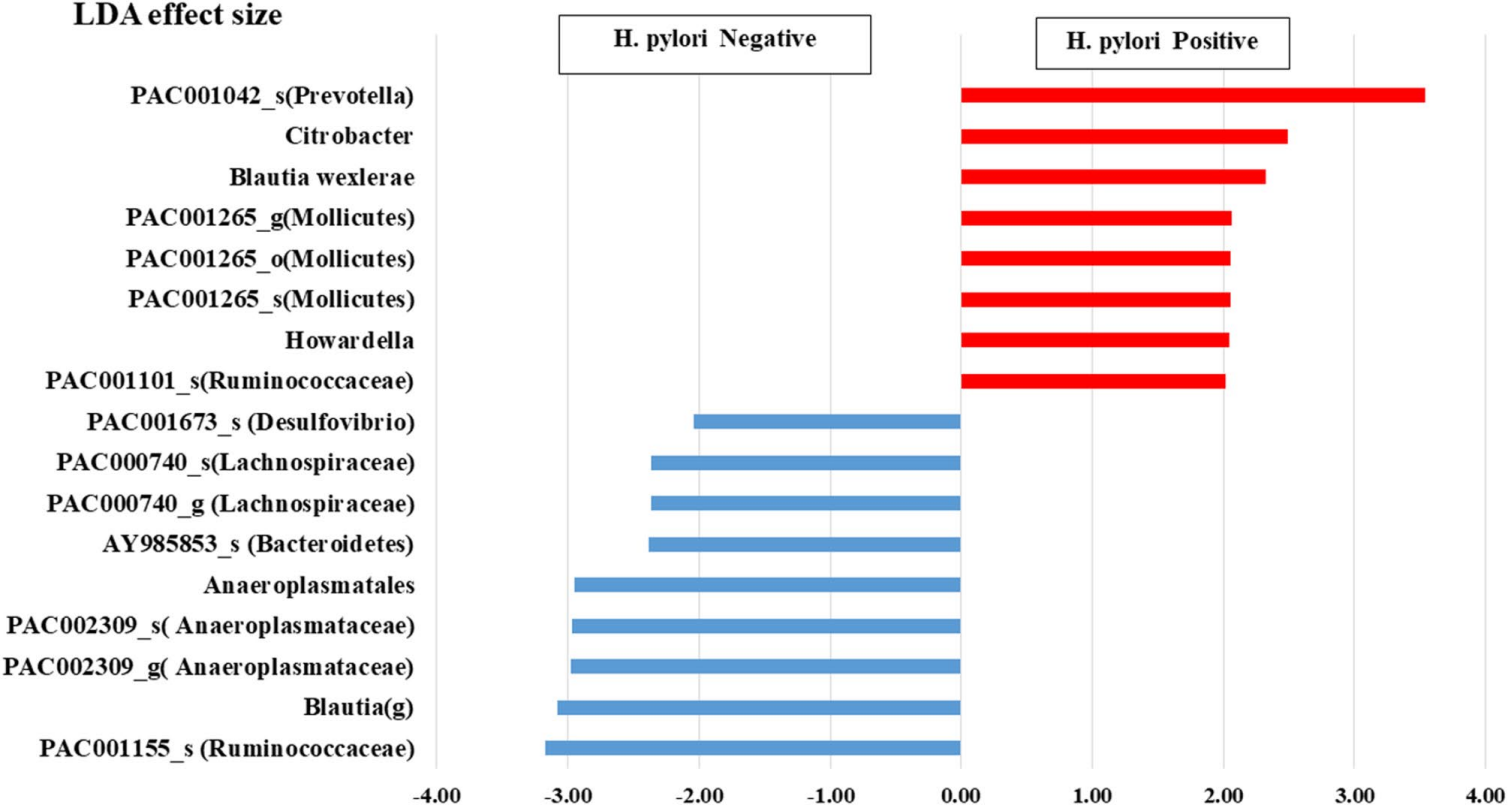
Taxonomic biomarker discovery LEfSe analysis. A bar plot demonstrating the relevant OTUs of the highest discrimination between *H. pylori*-positive (red bars) and -negative groups (blue bars) stratified in a descending order based on their logarithmic LDA score.

All taxa biomarkers with their differences in mean abundance between the *H. pylori*-positive and *H. pylori*-negative groups are listed in Supplementary Table 6. The LEfSe analysis in Fig. 2 displays the discriminatory taxa with the significant differences in mean abundance (FDR < 0.05) between both groups. The *H. pylori*-positive group exhibited distinctive existence of the following taxa in relation to LDA effect size: *PAC001042* species (Bacteroidetes: *Prevotella*), *Citrobacter* genus (Proteobacteria: *Enterobacteriaceae*), *Blautia wexlerae* spp. *(Clostridia: Lachnospiraceae)*, PAC001265 order (Tenericutes: *Mollicutes*), *Howardella (Clostridia: Lachnospiraceae)*, *PAC001101* spp. (*Clostridia: Ruminococcacea*e). The *H. pylori*-negative group exhibited a notable presence of *PAC001155_s* (*Clostridia*: *Ruminococcaceae*), *Blautia* genus, *PAC002309* (Tenericutes, *Mollicutes*), Anaeroplasmatales order (Tenericutes: *Mollicutes*), *AY985853_s* (Bacteroidetes: *Butyricimonas*), PAC000740_g (*Clostridia: Lachnospiraceae)*, PAC001673_s (Proteobacteria: *Desulfovibrio*).

### Gut microbial diversities

**Fig. 3:**
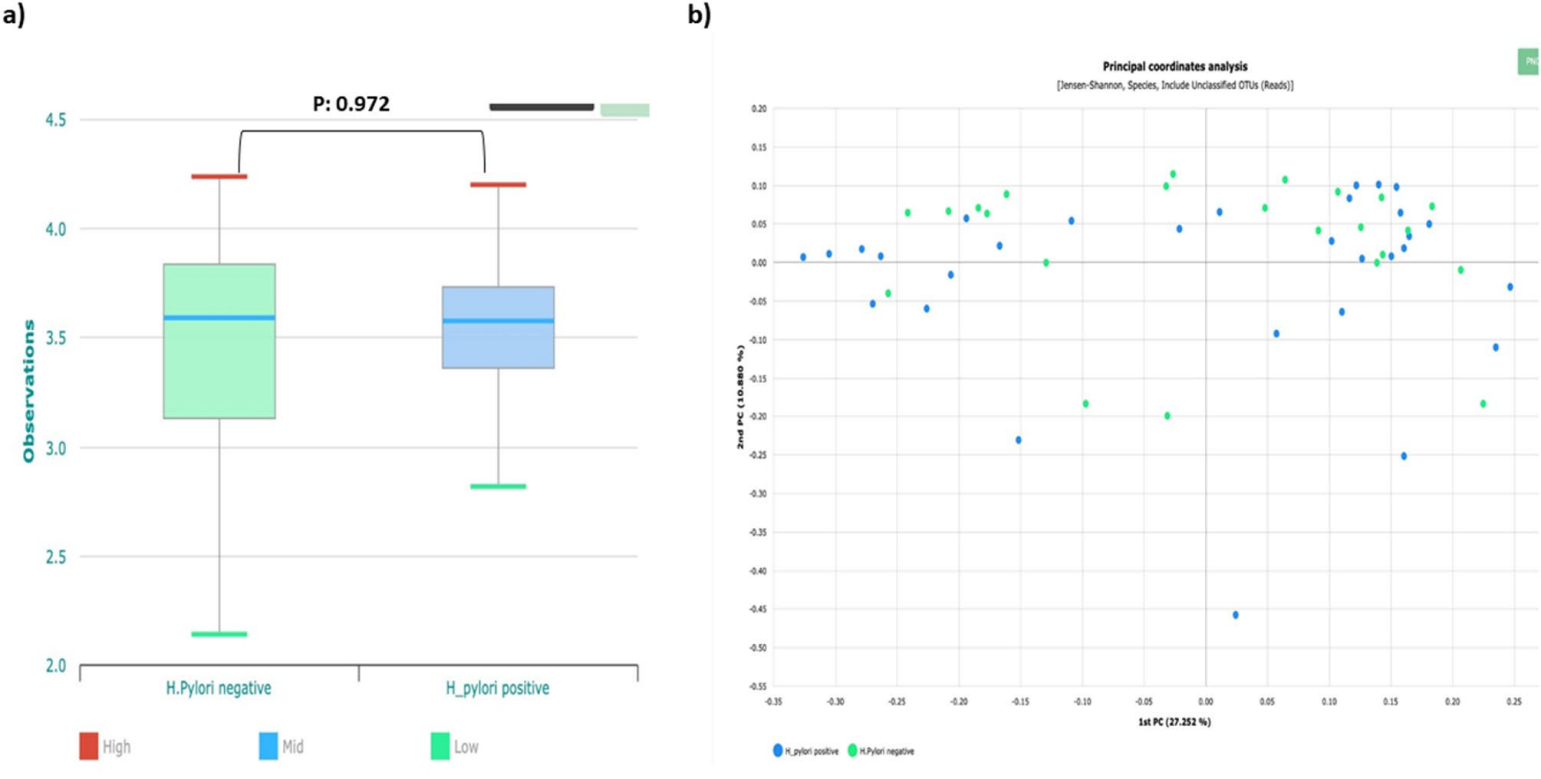
Comparative gut microbiota diversities between *H. pylori*-positive and –negative groups. **(a)** Alpha diversity: Boxplot depicting the difference in the Shannon index between both groups, **(b)** Beta diversity: principle coordinate analysis (PCoA) plot representing the extent of resemblance of microbiota profiles between both groups, where each coordinate displays the percent of variance. The Beta diversity distance was determined as per the Bray-Curtis dissimilarity index.

The alpha diversity of gut microbiota as determined by the Shannon index showed non-significant difference between *H. pylori*–positive and –negative groups (*p* value = 0.971). As depicted in the plotted two-dimensional principal coordinate analysis (PCA), the beta diversity represented by the Bray-Curtis dissimilarity index, revealed shared overlapping clusters of microbiota with subtle differences between both groups (Fig. 3).

**Fig. 4:**
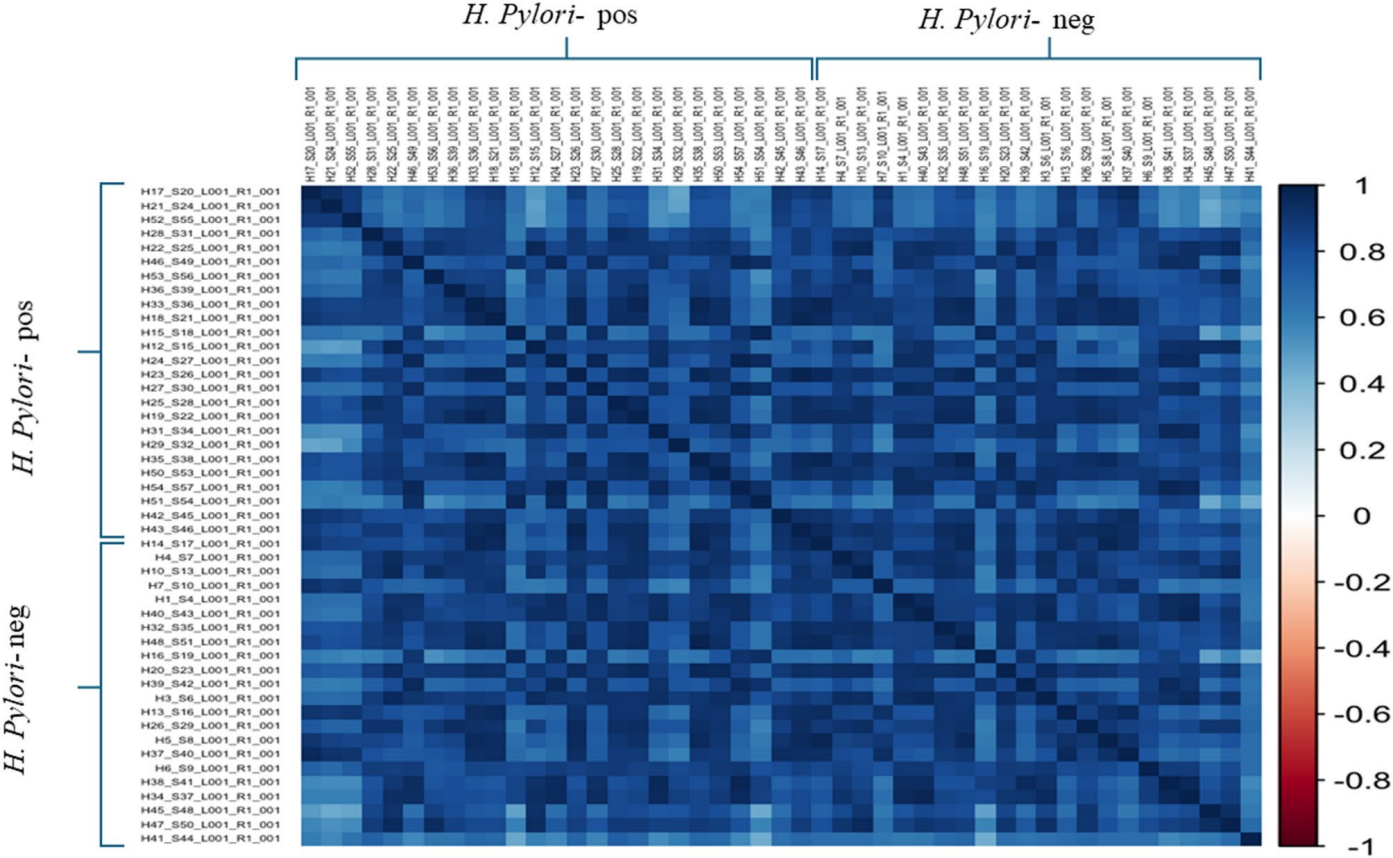
A Correlation heat map plot displaying clustering patterns of gut microbiota profiles among sequenced samples of *H. pylori*-positive and *H. pylori*-negative study groups. Samples ID are designated on both axes. The correlation scale extends from − 1 (highest negative correlation: deep red) to + 1 (greatest positive correlation: deep blue. The samples exhibiting the highest correlation (deepest blue) indicate the highest resemblance in microbiome profiles, while those moderate correlation with (lighter blue) reflect transitional microbiome architectures among samples.

As visualized in Fig. 4, the correlation matrix reveals clusters of microbiome signatures within each group of the study samples. Numerous samples show predominance of strong to moderate positive correlations throughout the matrix, with observed high correlation within the samples of the *H. pylori*-positive group sharing distinct signature of microbiota compared to control group, however low correlation between samples of *H. pylori*-positive and *H. pylori*-negative groups.

### Functional prediction of gut microbiota

The hierarchical KEGG functional pathway classification indicated a predominance of four level one pathways across all samples, in the order of metabolism, cellular processes, environmental information processing, and human diseases. The *H. pylori*-positive group exhibited a significantly higher mean abundance of the Metabolism (FDR = 0.0417) and Environmental information pathways (FDR = 0.0463) compared to the *H. pylori*-negative group (Supplementary Fig. 3 and Supplementary Table 7).

At level two KEGG classification, functional pathways were inferred as follows: AA metabolism, Signal transduction, Infectious disease and Cell growth and death. Two pathways (*AA* metabolism and Signal transduction) showed significantly higher abundances in the *H. pylori*-positive group compared to the *H. pylori*-negative group (FDR = 0.041 and FDR = 0.046, respectively). The Cell growth and death pathway, although exhibited higher abundance in the *H. pylori*-positive group compared to the *H. pylori*-negative group, however it showed no statistical difference according to the adjusted *p* value (FDR = 0.0606). In contrast, the Infectious disease pathway was more enriched in the *H. pylori*-negative compared to the *H. pylori*-positive group (Supplementary Fig. 4 and Supplementary Table 7).

**Fig. 5:**
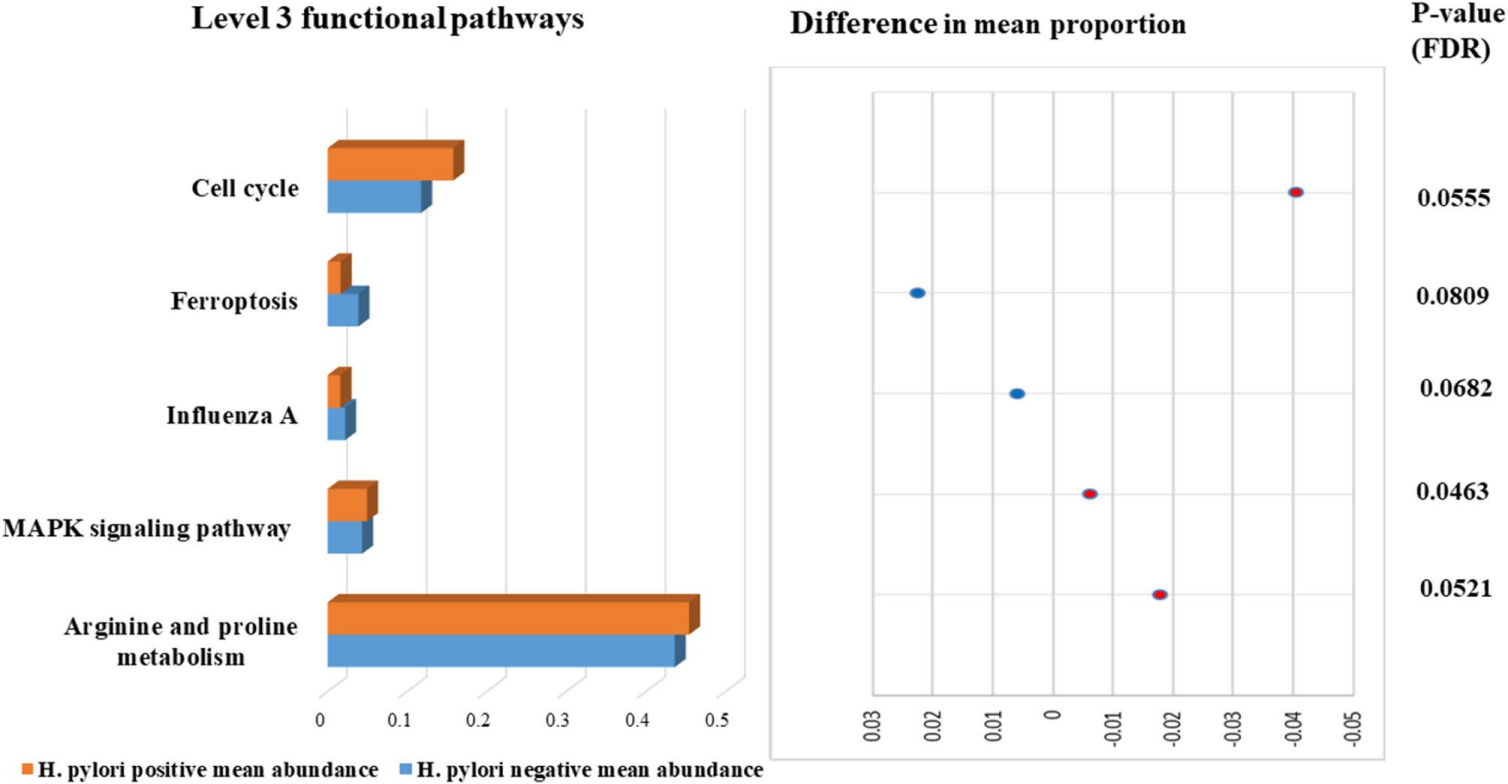
Comparative relative abundances and difference in mean proportions of level three gut microbiota KEGG functional pathways between *H. pylori*-positive and -negative groups.

**Fig. 6:**
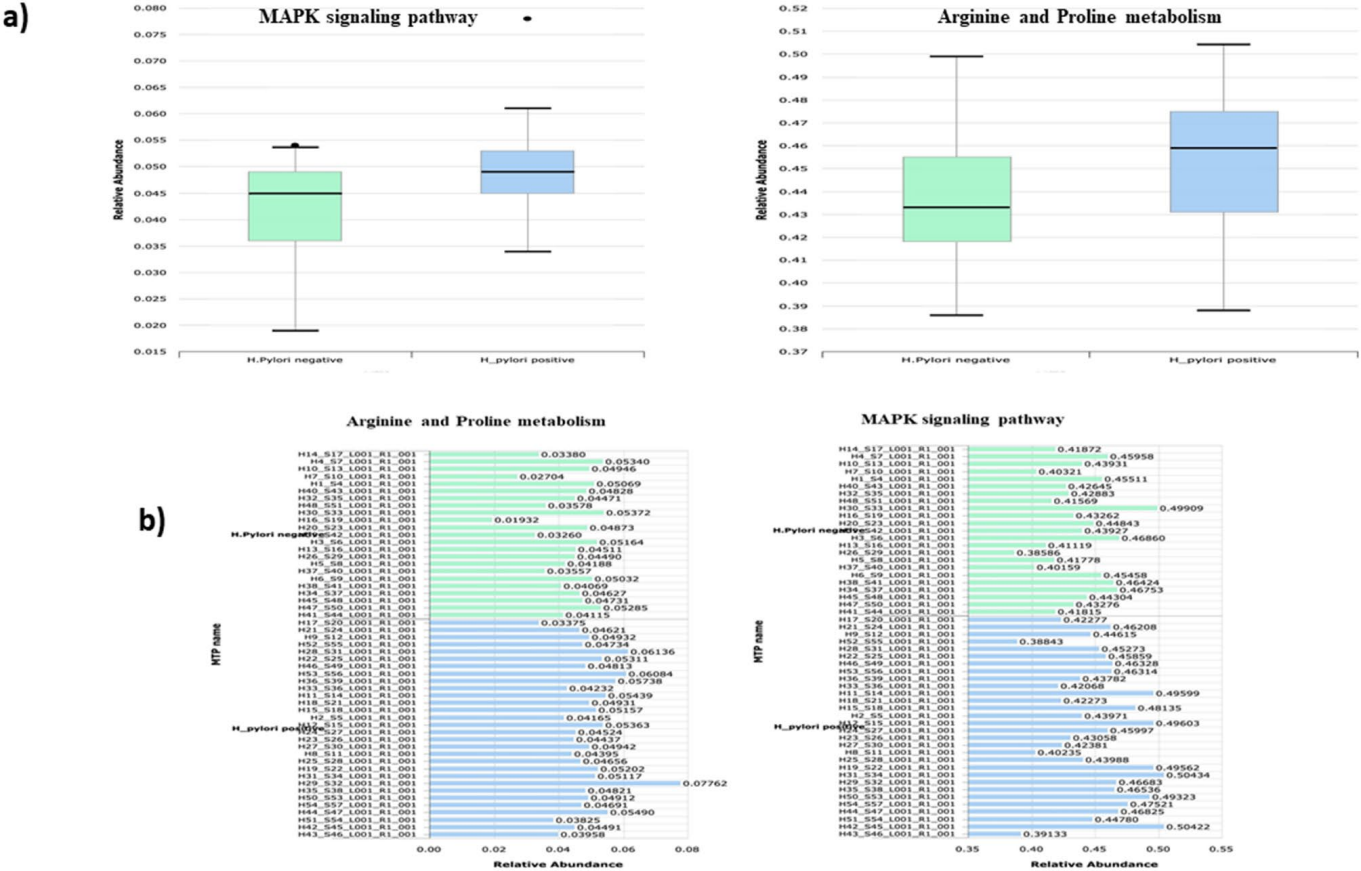
Mean abundance of level three functional pathways of gut microbiota among *H. pylori*-positive and -negative groups. (**a**) box plots representing higher average abundance of MAPK signaling and Arginine and proline pathways in *H. pylori*-positive than -negative group, (**b**) stacked bar representing the distribution of MAPK signaling and Arginine and proline pathways among individual samples of both groups.

Further analysis through level three KEGG pathways inferred five functional pathways ranked as follows: Arginine and proline metabolism, Cell cycle, MAPK signaling transduction, Ferroptosis and Influenza A. The first three predicted pathways were more enriched in the *H. pylori*-positive group, where only the MAPK signaling pathway exhibited a significant difference between both groups according to the adjusted *p* value (FDR = 0.0463). In contrast, Ferroptosis and Influenza pathways were predicted at non- significant lower abundance in the *H. pylori*-positive compared to the *H. pylori*-negative group (Figs. 5 and 6 and Supplementary Table 7). Using the KEGG Orthology (KO) database, four orthologues were recognized to be related to the predicted functional pathways as follows: (1) K16180 (methyl ornithine synthase) in Arginine and proline metabolism pathway, (2) K00940 (nucleoside-diphosphate kinase) in MAPK signaling pathway, (3) K01349 (furin) in Influenza A pathway, and (4) K13869 (solute carrier family-7) in Ferroptosis pathway.

## Discussion

*H. pylori* bacteria primarily colonizes the stomach mucosa, profoundly altering stomach microbiota composition, as evidenced in prior studies. However, its distal effects on intestinal tract microbiota and associated chronic intestinal disorders or colorectal malignancies remain understudied^5,11,20^. This study employed 16SrRNA sequencing to examine the alterations in gut microbiota among *H. pylori*-positive patients compared to controls. The taxonomic analysis of both groups indicated that phyla of Firmicutes, Bacteroidetes, Proteobacteria and Actinobacteria constitute the core of microbial communities in the gut^3^, aligning with the majority of previous reports on taxonomical profiles of gut microbiota^13,21^. Our study found that Proteobacteria were relatively more represented in the *H. pylori*-positive group, consistent with prior reports on *H. pylori*-infected cases, as *H. pylori* is recognized as a primary member of the Proteobacteria phylum^13,20^. Similarly, the Bacteroidetes phylum was found to be more prevalent in *H. pylori*-positive cases compared to controls, consistent with previously published data^22,23^. This finding is likely attributed to the override of the genera Bacteroides and Prevotella. In contrast, Actinobacteria and Firmicutes phyla were more abundant in *H. pylori*-negative group, emphasizing their significant role in health state. Actinobacteria includes the Bifidobacterium species, a well-known beneficial component of gut microbiota. Additionally, Firmicutes has been previously identified as the predominant core phylum of gut microbiota in healthy individuals^23^. These findings can infer the reduced (F/B) ratio in *H. pylori*-positive group compared to the negative group observed in our study, aligning with existing literature and underscoring the *H. pylori*-mediated alteration in microbiota composition^21,24^. Conversely, one study indicated a higher F/B ratio in *H. pylori*-positive cases, potentially due to differing criteria within the study population^25^.

The predominant genera in both groups were *Bacteroides* and *Prevotella*, with higher RA in the *H. pylori*-positive group. This aligns with a German study where *Prevotella* was more enriched in *H. pylori*-positive individuals, though *Bacteroides* predominated in controls^18,23^. Furthermore, our study displayed a relatively higher abundance of *Escherichia* genus in *H. pylori*-positive cases. The over-representation of *Prevotella* and *Escherichia* genera in *H. pylori* infection is corroborated by multiple studies in the literature^11,21^. *Prevotella* is a significant taxon within the oral cavity and esophagus of healthy individuals. The increased existence of *Prevotella* in the gut of *H. pylori*-infected patients, alongside other intestinal microbiota alterations, may stem from *H. pylori*-induced reductions in gastric acidity. Low stomach pH enables these acid-susceptible bacteria, to survive gastric transit and colonize the distant intestinal tract^5,14^. Interestingly, the gut predominance of *Prevotella* in *H. pylori* infection is considered a significant shift in *H. pylori*-mediated microbiota and together with other microbiota disturbances can trigger inflammatory responses, and elevate the risk of *H. pylori*-associated colorectal malignancy^18^. *Prevotella* is implicated in intestinal/extra-intestinal disorders and has been marked among colorectal malignancy-associated taxa^5,11,21^. In the same context, *E. coli* is involved in nitrite metabolism, leading to the generation of N-nitroso compounds that contribute to mucosal inflammations and carcinogenesis^14,26^. Consistent with earlier findings, our study showed a relative surpass of various genera in *H. pylori*-positive cases compared to controls including *Clostridia*, *Lactobacillus*, *Eubacteria* and *Alloprevotella*^10,21,25,27^. While *Lactobacillus* is known of its protective role in enhancing the human gut barrier; however, its prevalence in *H. pylori* infection remains contentious in the literature and differs between gastric and gut samples^3,28^. In the current study, the genus *Megasphaera* exhibited a relatively higher abundance in *H. pylori*-positive cases than in controls. This contrasts with a prior study reporting a higher abundance of *Megasphaera* levels in *H. pylori*-negative individuals, particularly in Chinese children with gastritis^21^.

On the other hand, *H. pylori*-negative group demonstrated proportional enrichment in genera of *Faecalibacter*, *Lachnospira*, *Alistipes* and *Bifidobacteria*. These genera are identified as short-chain fatty acid- producing microbiota that contributes to intestinal homeostasis and human gut health^5,14,29^. Therefore, their reduced abundance may confer negative repercussions on the host^23^. *Alistipes*, a relatively new-described genus in 2003, has been reported of its protective role against Gut Inflammatory disorders, where its dysbiosis may confer emerging implications to human health^30,31^. Nonetheless, there are conflicting reports concerning *Alistipes* and their association with multiple organ malfunctions, including those in the liver and cardiovascular systems^32^.

At the species level, a higher RA of *Bacteroides* species was observed in the *H. pylori*-positive group compared to the negative group, including *B. vulgatus and B. massiliense.* According to literature, some *Bacteroides* species that are part of the normal gut microbiota have been reported to be associated with specific disorders through pro-inflammatory effects^33–35^. The *H. pylori*-negative group exhibited an apparent predominance of other species, notably *Faecalibacter prausnitzii*, a short-chain fatty acid (SCFA)-producing bacterium that is recognized to play a crucial role in maintaining intestinal homeostasis and has been suggested for application among the next-generation probiotics (NGP)^34,36^.

The LEfSe analysis determined the most significant discriminatory taxa between *H. pylori*-positive and -negative groups. Our study revealed a significant override of *Prevotella* species (*PAC001042_s*), which aligns with previous reports which acknowledged *Prevotella* among microbiota shifts in *H. pylori* infection, and highlighted their role in intestinal disorders (10,11,18, 21). Additionally, the *H. pylori*-positive group exhibited distinctive representation of *Howardella*, which was consistent with a prior study indicated an increased prevalence of *Howardella* in patients infected with *H. pylori*^23^. *Howardella*, like *H. pylori*, produces urease, and its altered abundance has been associated with extra-intestinal disorders, including chronic renal damage attributed to ammonia release that adversely affects kidney interstitial tissue^37^. Furthermore, *Citrobacter* appeared as one of the discriminatory taxa in *H. pylori*-positive cases, in line with previous reports that noted associated high abundance of *Citrobacter* with *H. pylori* patients having gastric carcinoma^38,39^. On the other hand, our *H. pylori*-negative controls were distinguished by the over-enrichment of *Blautia* genus, *which* is classified within the *Lachnospiraceae* family and is naturally present in the gut, engaging in mutual interactions with the intestinal microbiome. There is an increasing evidence regarding its capacity to alleviate intestinal inflammation and metabolic disorders through the production of secondary bioactive metabolites that confer probiotic properties; however, further studies are necessary for a more comprehensive understanding^40^.

Our study noted relative predominance of *Desulfovibrio*, *Enterococcaceae*, *Rikenellacae*, and *Akkermansia* in the *H. pylori*-positive group compared to controls. Despite the low abundance of these taxa (RA < 1%), they have been recognized in the literature as clinically relevant regarding intestinal microbial shifts during infection^5^. Of interest, *Desulfovibrio* a sulfur- reducing bacteria found in the distal colon, may act as an opportunistic pathogen. Its overgrowth has been adversely associated with inflammatory bowel diseases (IBD), and even colon malignancies, due to DNA damage by H2S production^41^. Likewise, *Enterococcaceae*, *Rikenellaceae* and *Akkermansia* have been reported among *H. pylori*-altered gut taxa that contribute to intestinal barrier disruptions and subsequent colorectal malignancies^5,42,43^.

In agreement to previous reports, both *H. pylori*-positive and -negative groups in our study revealed comparable alpha and beta diversities of gut microbiota with no significant differences, denoting overall shared microbial communities between both groups^2,21^. Non-significant disparities in microbial diversities between both groups irrespective to the *H. pylori* state may be attributed to various explanations including relatively low study samples. Additionally, the possibility of a recent *H. pylori* infection among enrolled *H. pylori*-positive patients may not allow a sufficient time for relevant deviations in microbial diversities to emerge between *H. pylori*-positive group and controls. Nevertheless, our study demonstrated higher correlation in gut microbiome profile within the samples of *H. pylori*-positive group, than between those of *H. pylori*-positive and *H. pylori*-negative groups, suggesting that *H. pylori* status implies unique signature on gut microbiota composition.

Numerous studies have emphasized the crucial impact of *H. pylori* on the gut microbial community^5^, and its crosstalk with other gut bacteria. *H. pylori* induces gut microbial dysbiosis through multiple mechanisms, including alterations in gastric pH, provision of substrates that facilitate bacterial colonization, and modulation of host dietary and lifestyle habits^13^. Notably, the eradication regimen itself significantly disrupts gut equilibrium, underscoring the need for probiotic supplementation to preserve microbial ecology during treatment^35^. *H. pylori*-mediated intestinal dis-homeostasis culminates in chronic digestive disorders; furthermore, it may substantially contribute to tumour genesis due to persistent inflammation and toxic metabolites^13,44^. Noteworthy, a variety of confounding factors can impact the composition of the intestinal microbiota, such as aging, dietary habits, geographical population, genetic predisposition, antibiotic use, and socioeconomic factors. These factors either foster microbial diversity and supports beneficial bacteria, or promote dysbiosis through proliferation of pathogenic species^45^.

Through functional analysis of gut microbiota in both study groups using KEGG databases, our study predicted core functional pathways related to aa metabolism, cell growth and death, signal transduction and infectious diseases. A deeper insight to level three functional pathways inferred significant predominance of the MAPK signaling transduction pathway in *H. pylori*-positive group compared to the controls. According to literature, MAPK signaling and Cell death pathways are notably interrelated in *H. pylori*-positive cases. Under physiological conditions, programmed cell death (PCD) acts as an essential process to maintain homeostasis in human Body^46^. Pathogens like *H. pylori* infection can disrupt this process and potentially lead to oncogenesis. Recent literature indicates that *H. pylori* can activate the MAPK signaling pathway, which influences cell growth, differentiation, and apoptosis, potentially resulting in malignant transformation, referred to as the “Cancer-linked signaling pathway”^46,47^. Furthermore, *H. pylori* can enhance the expression of PCD-ligand on infected epithelial cells, which shields the infected cells from the human immune response and preventing cell death^46,48^. Furthermore, our study observed relative elevated levels of the Arginine and proline metabolism pathway, along with the Cell cycle and death pathway in *H. pylori*-positive cases; however, these findings were not statistically significant when compared to the control group. Our findings are consistent with a recent study indicating a predominance of functional pathways related to amino acid and carbohydrate metabolism, cell cycle, and apoptosis in patients infected with *H. pylori*^2^. A separate study identified diverse pathways related to sugar and fatty acid metabolism; however, it is important to note that it focused on the Chinese population^18^. The Arginine and proline metabolism pathway can play a vital role in mediating local and systemic human immune response^49^. Understanding the function of this pathway in gut microbial ecology may elucidate its increased prevalence in *H. pylori* infection. Arginine regulates the production of nitric oxide, urea, and ornithine, and supports the metabolism of sugars and fats, whereas proline mediates cellular energy supply, redox and signaling processes^49,50^. Arginine acts as a substrate for two enzymes: arginase, which produces urea and ornithine, and nitric oxide synthase, which generates nitric oxide (NO)^51^. *H. pylori* induces the production of the arginase enzyme, which facilitates its survival by competing with nitric oxide synthase for arginine, thereby decreasing nitric oxide production, which is bactericidal to *H. pylori*^51^. In other way, Arginase generates urea, serving as a source of ammonia required by *H. pylori* to increase gastric pH and produce ornithine, inhibiting NO synthase^52^. The proline may functionally engage with arginine, as the ornithine metabolite of arginine can generate the precursor of proline through the action of ornithine aminotransferase^53^. Some of these metabolic reactions play a role in the pathogenicity of *H. pylori*. Specifically, spermine, a metabolite of ornithine, is oxidized by spermine oxidase, resulting in the production of hydrogen peroxide, which leads to oxidative stress, cell apoptosis, and DNA damage associated with *H. pylori*^52^. Conversely, it can be argued that the reduced production of NO due to *H. pylori* may guard against the inflammatory effects of NO, which leads to TNF-dependent neutrophil infiltration of the mucosa^51^. This finding may support the proposed the unpopular opinion that the presence of *H. pylori* may protect against inflammatory bowel diseases^35^. Consequently, in addition to the notable changes in microbiota associated with *H. pylori* eradication antimicrobial regimens^3^, alternative approaches have surfaced, suggesting that not every *H. pylori*-positive case necessitates eradication^35^. It is intriguing to note that ariginine and proline along with glutamine constitute arginine-proline- glutamine metabolic axis, which has been implicated in cancer growth and proliferation^50^.

On the other hand, our study inferred a reduced abundance of the Ferroptosis pathway in *H. pylori-positive* cases, when compared to the controls. A recent publication offered an explanation that *H. pylori* promotes the upregulation of the cystine/glutamate antiporter subunit “Solute Carrier Family 3 Member 2” (SLC3A2) and concurrently downregulates “transferrin receptor 1” (TfR1), which is essential for iron uptake, thereby inhibiting Ferroptosis^54,55^. Nevertheless, other reports suggest hat *H. pylori* may induce Ferroptosis by influencing specific Ferroptosis-associated genes^51,56^, denoting that *H. pylori* may activate or attenuate Ferroptosis^46^. Recent evidence indicates that Cell death pathways are activated during the acute phase of *H. pylori* infection, resulting in mucosal damage to the stomach. In contrast, during the chronic phase, these pathways become attenuated, which may promote the development of malignant tumours^46^. These findings infer that the microbiota’s functional pathways interact systematically with the human host, affecting both physiological processes and pathological conditions.

The findings of this study highlighted significant compositional and functional variations in gut microbiota between *H. pylori*-positive cases and -negative controls. Our study emphasized the discriminatory gut taxa associated with *H. pylori* and their clinical significance, as supported by prior studies. Additionally, we explored the predicted functional pathways of gut microbiota in *H. pylori-positive* cases that could be implicated in *H. pylori*-associated gut disorders. This study presents the inaugural report on the predicted functionality of the *H. pylori-*associated gut microbiome in a Middle-Eastern country. Nonetheless, our study was constrained by a relatively modest sample size. Furthermore, it is important to emphasize that the reported functional pathway alterations represent inferred predictions based on 16SrRNA data rather than whole metagenome or metatranscriptome sequencing, and the biological significance remains speculative. Future studies should consider incorporating further complementary meta-transcriptomic or metabolomic methodologies to substantiate predicted functional shifts and provide stronger mechanistic insights into microbiome alterations associated with *H. pylori* infection.

Enhanced understanding of gut microbiota-mediated functional processes can bring new avenues for innovative *H. pylori* treatment approaches that may supplement or replace the existing eradication regimens and their associated sequelae of microbial dysbiosis. In addition, they may also be effective in alleviating the risk of *H. pylori*-induced development of gastric or colorectal carcinoma.

## Conclusion

The *H. pylori*-positive group in our study exhibited a relative override of Bacteroidetes and Proteobacteria phyla, mainly genera of *Bacteroides*, *Prevotella* and *Escherichia*, along with species including *Bacteroides vulgatus* and *E. coli* compared to the controls. The significant discriminatory taxa in *H. pylori*-positive cases included *Prevotella* species (*PAC001042_s)*,* Citrobacter* and *Howardella*, which were previously reported of their positive association with inflammatory responses and gastrointestinal disorders. *Desulfovibrio*, *Enterococcaceae*, *Rikenellaceae* and *Akkermansia* exhibited relatively higher abundance in the *H. pylori*-positive group compared to controls. While these taxa were observed in low abundance, they have been previously recognized for their clinical significance concerning metabolic disorders, inflammatory bowel diseases, and colon malignancies. *H. pylori*-negative controls exhibited significant higher abundance of *Blautia* along with relative over-representation of *Alistipes* compared to controls, where both taxa are marked as protective components of gut microbiome. Comparative microbial diversities suggest overlapping gut microbiome structure between *H. pylori*-positive and-negative groups. However, the more pronounced correlation in gut microbiome profile within *H. pylori*-positive group, than between *H. pylori*-positive and *H. pylori*-negative groups infers that *H. pylori* status implies a unique signature on gut microbiota composition.

Functional prediction of gut microbiome inferred pathways in the form of AA metabolism, Cell growth and death, Signal transduction and Infectious diseases. At level three functional pathways, MAPK signaling transduction pathway was inferred at significant higher abundance in *H. pylori*-positive group compared to the control group. Furthermore, Arginine and proline metabolism along with Cell cycle and death pathways showed relatively higher prediction in *H. pylori*-positive cases, when compared to controls, however with non- significant difference between both groups. Literature insights into the capabilities of these functional pathways reveal their contributing role in the pathogenicity of *H. pylori*, by mediating oxidative stress, cell apoptosis and DNA damage, delineating their implication in malignant transformation and cancer development. Enhancing the comprehension of these predicted functional potentials of *H. pylori*-altered gut microbiome through further multi-omics researches may revolutionize therapy by developing inventive strategies that complement current eradication regimens, thereby addressing microbial dysbiosis and mitigating the risk of adverse complications.

## Supplementary Information

Below is the link to the electronic supplementary material.

Supplementary Material 1

## Data Availability

The datasets generated and/or analysed during the current study are available in the NCBI, under *H. pylori* associated gut microbiota Bio-project number: PRJNA1252884.https://dataview.ncbi.nlm.nih.gov/object/PRJNA1252884?reviewer=1fo23fg1mms7dkfn9roagfi6gk.
