# Supplementary material for: Exploring compositional and predicted functional alterations of gut microbiota in H. pylori infection
Source: Sci Rep. 2025 Oct 29;15:37745. doi: 10.1038/s41598-025-22788-4 (PMC12572280; doi:10.1038/s41598-025-22788-4)

Supplementary Table 1: Valid sequence reads of Microbiome taxonomic profiles across samples of the two groups

Sample name	Target reads	OTUs	ACE	Shannon	Good's coverage (%)
H14_S17_L001_R1_001	16224.00	172	220.96	3.05	99.72
H4_S7_L001_R1_001	26657.00	365	423.32	4.06	99.73
H17_S20_L001_R1_001	5823.00	84	114.11	2.61	99.61
H10_S13_L001_R1_001	29713.00	312	352.01	3.88	99.82
H21_S24_L001_R1_001	15305.00	144	183.10	2.88	99.76
H7_S10_L001_R1_001	34420.00	123	158.59	2.14	99.90
H1_S4_L001_R1_001	20265.00	281	340.17	3.72	99.69
H40_S43_L001_R1_001	41074.00	381	444.20	3.75	99.82
H52_S55_L001_R1_001	43878.00	195	223.02	2.82	99.92
H28_S31_L001_R1_001	1303.00	147	218.34	3.79	95.55
H32_S35_L001_R1_001	47436.00	270	314.57	3.59	99.89
H22_S25_L001_R1_001	29655.00	327	361.22	4.06	99.84
H49_S52_L001_R1_001	23431.00	320	399.95	3.72	99.65
H46_S49_L001_R1_001	39354.00	353	400.47	3.36	99.85
H48_S51_L001_R1_001	45301.00	298	336.07	3.28	99.89
H53_S56_L001_R1_001	32019.00	381	443.81	3.64	99.77
H36_S39_L001_R1_001	41093.00	311	395.53	3.51	99.80
H30_S33_L001_R1_001	16591.00	222	256.77	3.18	99.73
H33_S36_L001_R1_001	33751.00	297	373.05	3.66	99.78
H16_S19_L001_R1_001	18021.00	151	187.42	2.38	99.79
H18_S21_L001_R1_001	15002.00	244	291.98	3.73	99.64
H20_S23_L001_R1_001	15061.00	273	335.00	4.06	99.58
H39_S42_L001_R1_001	41456.00	408	467.47	3.02	99.81
H15_S18_L001_R1_001	13586.00	204	281.19	3.37	99.57
H3_S6_L001_R1_001	28894.00	439	534.01	4.12	99.63
H12_S15_L001_R1_001	27483.00	397	469.47	4.36	99.73
H38_S41_L001_R1_001	34567.00	400	441.44	3.67	99.82
H24_S27_L001_R1_001	44321.00	420	474.56	3.72	99.84
H13_S16_L001_R1_001	28760.00	301	345.17	3.55	99.82
H23_S26_L001_R1_001	46546.00	292	343.45	3.56	99.88
H26_S29_L001_R1_001	19220.00	275	341.17	3.69	99.65
H27_S30_L001_R1_001	40761.00	398	458.20	3.48	99.81
H5_S8_L001_R1_001	25483.00	253	306.49	3.32	99.79
H25_S28_L001_R1_001	18975.00	370	456.55	4.03	99.51
H19_S22_L001_R1_001	14272.00	280	372.30	3.99	99.46
H31_S34_L001_R1_001	45883.00	354	390.72	3.67	99.89
H37_S40_L001_R1_001	48826.00	311	366.67	3.09	99.86
H6_S9_L001_R1_001	24055.00	217	271.64	3.43	99.78
H29_S32_L001_R1_001	37693.00	252	294.03	3.61	99.87
H35_S38_L001_R1_001	47378.00	308	345.20	3.62	99.89
H34_S37_L001_R1_001	36175.00	385	448.79	3.81	99.79
H45_S48_L001_R1_001	39891.00	394	483.72	3.97	99.78
H50_S53_L001_R1_001	39583.00	489	532.16	4.20	99.83
H54_S57_L001_R1_001	28345.00	385	452.91	3.64	99.71
H44_S47_L001_R1_001	30939.00	220	285.48	1.07	99.78
H47_S50_L001_R1_001	40721.00	491	562.70	4.24	99.78
H41_S44_L001_R1_001	33627.00	183	218.99	2.51	99.88

H51_S54_L001_R1_001	43595.00	327	367.55	2.94	99.88
H42_S45_L001_R1_001	35950.00	229	271.24	3.47	99.87
H43_S46_L001_R1_001	41561.00	249	315.74	3.50	99.86

Supplementary Table 2: The mean abundance of main gut microbiome taxa in the study

Taxonomy level	Main taxa with the highest RA (>1%)	Average relative abundance (%)		
		H. pylori-pos (95% CI)	*H. pylori*-neg (95% CI)	P-value
Phyla	Firmicutes	45.91% (41.23-50.19)	48.82% (43.86-53.77)	0.451
	Bacteroidetes	43.59% (38.11-49.02)	41.02% (35.71-46.32)	0.473
	Proteobacteria	8.36% (2.54-14.57)	7.69% (2.73-12.65)	0.971
	Actinobacteria	1.07% (0.60-1.55)	1.33 % (0.57-2.08)	0.430
Genera	Bacteroides	22.55% (14.80-29.74)	22.10% (14.48-29.70)	0.747
	Prevotella	13.48% (6.69-21.15)	10.90% (4.39-17.41)	0.554
	Faecalibacterium	6.39% (4.70-7.46)	7.17% (5.37-8.95)	0.379
	Escherichia	4.19 % (-1.089-9.71)	1.97 % (-0.21-4.15)	0.092
	Dialister	3.69 % (2.28-5.26)	3.88 % (2.60-5.14)	0.554
	Lachnospira	1.86 % (1.18-2.53)	3.09 % (1.45-4.72)	0.399
	Clostridium_g24	1.68% (0.72-2.71)	1.26% (0.65-1.87)	0.196
	Lactobacillus	1.59 % (0.12-3.16)	1.25 % (0.26-2.22)	0.760
	Oscillibacter	3.12 % (1.95-4.25)	3.49 % (2.32-4.65)	0.440
	Eubacterium_g23	2.16 % (1.15-3.30)	1.63 % (1.00-2.26)	0.957
	Alloprevotella	2.07 % (0.73-3.55)	1.85 % (-0.32-4.03)	0.190
	Alistipes	1.79 % (1.01-2.42)	2.22 % (1.20-3.24)	0.409
	Megasphaera	1.58% (0.39-2.78)	0.62% (-0.08-1.32)	0.320
Species	Bacteroides vulgatus	6.66% (6.65-6.68)	4.6% (1.84-7.42)	0.282
	Faecalibacterium Prausnitzii	5.5% (5.42-5.43)	6.36% (4.70-8.03)	0.399
	Prevotella PAC001304_s	5.5% (5.15-5.53)	6.59% (1.26-11.92)	0.936
	Prevotella_uc	3.86% (3.85-3.87)	2.43% (0.302-4.57)	0.936
	Bacteroides uniformis	3.56% (3.55-3.57)	4.8% (0.93-8.72)	0.490
	Bacteroides dorei	3.17%(3.16-3.18)	5.09% (1.57-8.62)	0.146
	Escherichia coli	4.3%(4.29-4.32	1.97% (-0.21-4.15)	0.092
	Dialister PAC001039_s	2.00%(1.99-2.01)	1.34% (0.40-2.28)	0.943

CI: Confidence Interval, **RA**: Relative abundance

Supplementary Table 3: Microbiome taxonomic composition at the phylum level

	H. pylori positive	H. pylori negative
Proteobacteria	8.36	7.69
Actinobacteria	1.07	1.33
Bacteroidetes	43.59	41.02
Firmicutes	45.91	48.82
Verrucomicrobia	0.8683	0.7215
Synergistetes	0.0034	
Fusobacteria	0.0071	0.0086
Cyanobacteria	0.0828	0.0843
Lentisphaerae	0.0142	0.0078
Chloroflexi	0.0001	
Elusimicrobia	0.0020	0.0014
Saccharibacteria_TM7	0.0317	0.0324
Tenericutes	0.0592	0.2736

Supplementary Table 4: Microbiome taxonomical composition at the Genus level

	H. pylori -positive	*H. pylori* -negative
Subdoligranulum	1.98	2.10
Sporobacter	1.17	1.27
Escherichia	4.19	1.97
Eubacterium_g23	2.16	1.63
Oscillibacter	3.12	3.49
Roseburia	2.01	2.30
Dialister	3.69	3.88
Prevotella	13.48	10.9
Lachnospira	1.86	3.09
Alistipes	1.79	2.22
Bacteroides	22.55	22.1
Parabacteroides	1.88	1.79
Clostridium_g24	1.68	1.26
Megasphaera	1.54	0.6218
Alloprevotella	2.07	1.85
Agathobacter	1.68	2.33
PAC001046_g	1.09	1.63
Lactobacillus	1.59	1.25
Faecalibacterium	6.39	7.17
JX575929_g	0.0013	0.0002
PAC001212_g	0.0003	0.0042
GQ451199_g	0.0077	0.1953
FR893353_g	0.0092	0.0121
PAC001137_g	0.0091	0.0081
Rikenellaceae_uc	0.0005	0.0004
GQ448104_g	0.0007	0.0096
Proteus	0.0224	0.0011
Barnesiella	0.4152	0.5521
Veillonella	0.4839	0.1397
HQ400094_g	0.0435	0.0004
Lysinibacillus	0.0001	
PAC002374_g	0.1042	
Scardovia	0.0001	0.0009
Finegoldia	0.0008	0.0009
Desemzia	0.0001	
GU324393_g	0.0010	0.0028
PAC001269_g	0.1444	0.1046
Kluyvera	0.0004	
PAC001200_g	0.0049	0.0110
PAC000677_g	0.0031	0.0005
PAC000194_g	0.0059	0.0132
PAC000740_g	0.0378	0.0750
Pseudoflavonifractor	0.2856	0.3498
PAC002518_g	0.1014	0.0549
Kineothrix	0.0010	0.0002

Olsenella	0.0055	0.0100
PAC002155_g	0.0004	0.0009
Acidaminococcus	0.0604	0.1957
JX198636_g	0.0005	0.0002
NHOC_g	0.0023	0.0002
AB218327_g	0.0135	0.0002
PAC002309_g	0.0129	0.1897
DQ800447_g	0.0068	0.0027
EF404387_g	0.0098	0.0007
PAC001138_g	0.3473	0.4989
AB606281_g	0.0003	0.0003
PAC001115_g	0.2337	0.2647
Lactococcus	0.0117	0.0103
PAC001573_g	0.0001	0.0002
Rothia	0.0039	0.0045
AB559589_g	0.0039	0.0010
EU728715_g	0.0015	0.0025
PAC001247_g	0.0911	0.2153
PAC002143_g	0.0206	0.0005
Massilioclostridium	0.0024	0.0072
PAC001201_g	0.0109	0.0114
Faecalimonas	0.0008	0.0004
Ezakiella	0.0006	0.0003
Cardiobacterium	0.0002	0.0002
Desulfovibrionaceae_	0.0021	
PAC001236_g	0.0211	0.0446
Collinsella	0.1119	0.0670
Actinomyces	0.0065	0.0063
Moryella	0.0034	
Leuconostoc	0.0033	0.0312
FMWZ_g	0.0005	
PAC001606_g	0.0082	0.0033
GQ451241_g	0.0211	
PAC002169_g	0.0001	0.0002
Butyricicoccus	0.0004	
Cloacibacillus	0.0003	
OCTT_g	0.0014	0.0007
Blautia	0.3688	0.5984
Anaerococcus	0.0024	0.0005
Dielma	0.0002	
AF125206_g	0.0007	0.0003
AM500849_g	0.0005	0.0007
FR888536_f_uc	0.0008	0.0010
HQ748727_g	0.0007	0.0009
Anaerostipes	0.0362	0.0500
PAC001468_g	0.0373	0.0114

Turicibacter	0.2606	0.0186
PAC001422_g	0.0117	
PAC002409_g	0.0004	
Anaerofilum	0.0016	0.0012
HQ765927_g	0.0012	0.0003
Aerococcus	0.0001	
Murdochiella	0.0004	0.0017
Turicimonas	0.1214	0.0961
FJ681847_g	0.0001	0.0002
PAC001457_g	0.0043	0.0048
PAC002046_g	0.0039	0.0058
Acetitomaculum	0.0013	0.0036
Frisingicoccus	0.0775	0.1416
EU794292_g	0.0324	0.0945
PAC001313_g	0.0135	0.0118
Streptococcus	0.5540	0.4723
Mycoplana	0.0002	0.0003
Paracoccus	0.0001	
Chryseobacterium	0.0001	0.0002
PAC002156_g	0.0010	0.0015
Slackia	0.0014	0.0049
PAC001446_g	0.0164	0.0090
PAC001400_g	0.0001	
EU794101_g	0.0119	0.0029
Muribaculum	0.0035	0.0013
EU794124_g	0.0014	
PAC002148_g	0.0147	0.0101
AF349416_g	0.0019	
PAC001458_g	0.1319	0.0789
PAC000672_g	0.1263	0.1320
Terrisporobacter	0.0043	0.0025
LT706945_g	0.0234	0.0182
PAC001435_g	0.0362	0.0129
PAC001057_f_uc	0.0114	
Abiotrophia	0.0086	0.0011
Christensenella	0.0282	0.0186
Neisseria	0.0007	0.0003
PAC001447_g	0.0025	0.0041
PAC000661_g	0.6701	1.38
PAC001401_g	0.0084	0.0058
Eubacterium	0.0025	0.0002
LT575483_g	0.0171	
AJ518873_g	0.0041	0.0059
Enterobacter	0.2664	0.1062
PAC001603_g	0.0093	0.0232
Anaerotignum	0.2075	0.2228

PAC002509_g	0.0002	
PAC001592_g	0.0065	0.0032
Bilophila	0.1398	0.2010
AY858456_g	0.0009	0.0008
Hungatella	0.0218	0.0114
Enterobacteriaceae_u	0.0482	0.0319
Haemophilus	0.0887	0.3218
Longicatena	0.0055	0.0210
Enterococcaceae_uc	0.0002	
Merdimonas	0.0006	0.0017
Paludicola	0.0377	0.0337
PAC001270_g	0.0018	0.0029
Selenomonadaceae_	0.0002	
Ruminococcaceae_uc	0.0218	0.0138
PAC001637_g	0.0445	0.0252
Succinivibrio	0.4077	2.00
Merdibacter	0.0004	0.0013
Fenollaria	0.0010	0.0053
PAC001217_g	0.0026	0.0058
Libanicoccus	0.0001	0.0010
Salmonella	0.0008	
PAC001402_g	0.0006	0.0006
Holdemania	0.0146	0.0200
Helicobacter	0.0003	0.0001
Helcococcus	0.0001	
FJ366802_g	0.0003	
Howardella	0.0240	0.0022
Anaerotruncus	0.0191	0.0106
Elusimicrobium	0.0020	0.0014
Lachnoclostridium	0.0007	0.0019
PAC001109_g	0.0043	0.0024
PAC001625_g	0.0006	0.0005
Acutalibacter	0.0089	0.0082
PAC001207_g	0.5021	0.4210
PAC001437_g	0.0234	0.0089
EU137610_g	0.0002	0.0006
FJ534957_g	0.0003	0.0001
Porphyromonas	0.0016	0.0017
AB506418_g	0.0107	
Bacteroidaceae_uc	0.0137	0.0028
Romboutsia	0.0480	0.3917
Sneathia	0.0020	
CCYH_g	0.0032	0.0037
Coprococcus	0.2359	0.5109
Atopobium	0.0028	0.0041
Clostridium_g6	0.0060	0.0072

KE159605_g	0.0019	0.0033
PAC002201_g	0.0004	
Sutterella	0.3076	0.3010
Pseudobutyrivibrio	0.0001	
Cronobacter	0.0023	0.0026
PAC001283_g	0.0448	0.1446
EU775066_g	0.0003	
Acinetobacter	0.0039	0.0036
PAC000671_g	0.0151	0.0021
Muribaculaceae_uc	0.0029	0.0861
Alishewanella	0.0001	
FR888536_g	0.0714	0.0833
PAC001600_g	0.0061	0.0101
Sphingomonas	0.0001	0.0002
Pantoea	0.0075	0.0047
PAC001041_g	0.0081	0.0117
GQ451258_g	0.0023	0.0027
Marvinbryantia	0.0054	0.0038
Providencia	0.0021	
Pseudoxanthomonas	0.0002	
KE993550_g	0.0019	0.0041
Phocea	0.0098	0.0051
Comamonadaceae_u	0.0003	0.0063
PAC001219_g	0.0096	0.0117
PAC000683_g	0.0118	0.0002
Weissella	0.1404	0.2175
Lautropia	0.0001	0.0004
DQ807451_g	0.0003	0.0005
PAC001778_g	0.0050	0.0267
PAC001480_g	0.0329	0.0038
Gordonibacter	0.0009	0.0015
Metakosakonia	0.0004	0.0001
Eisenbergiella	0.3034	0.4180
Christensenellaceae_	0.0111	0.0076
Erwinia	0.0002	0.0001
PAC000748_g	0.1705	0.1725
Bacteroides_g1	0.0714	0.0121
Pluralibacter	0.0002	
Enterococcus	0.0024	0.0086
Mycobacterium	0.0001	
Cutibacterium	0.0002	0.0003
Megamonas	0.6550	0.0001
Anoxybacillus	0.0002	
PAC002129_g	0.0029	0.0008
Dehalobacterium_f_ι	0.0012	0.0021
PAC002423_g	0.0010	0.0027

AM405126_g	0.0023	0.0032
Morganella	0.1133	0.0002
Ruminococcus	0.9801	1.02
Enterobacteriaceae_g	0.9456	0.5910
PAC002170_g	0.0001	0.0002
PAC001296_g	0.0050	0.0055
Phascolarctobacteriu	0.8228	0.6977
Clostridium_g35	0.0061	0.0108
Clostridium_g34	0.0002	0.0002
Clostridium	0.1157	0.1789
Ruminococcus_g2	0.9480	1.17
Ruminococcus_g5	0.0656	0.0445
Ruminococcus_g4	0.2722	0.4498
Catenibacterium	0.1226	0.0332
PAC002203_g	0.0015	0.0053
Intestinibacter	0.0107	0.0127
Eggerthella	0.0030	0.0054
PAC001512_g	0.0014	
Desulfovibrio	0.1583	0.0283
PAC001360_g	0.0190	0.0240
PAC001032_g	0.1028	0.1886
PAC000692_g	0.1044	0.1227
EF025282_g	0.0007	0.0010
PAC001141_g	0.0251	
Kingella	0.0001	
Gemella	0.0107	0.0047
Saccharimonas	0.0279	0.0315
Peptoniphilus	0.0013	0.0022
FJ677212_g	0.0036	0.0015
AM278923_g	0.0002	0.0008
Serratia	0.0003	
AB730688_g	0.0001	
PAC001274_g	0.0013	0.0032
DQ824211_g	0.0512	0.0276
PAC001043_g	0.2763	0.3525
DQ777889_g	0.0014	0.0103
LARJ_g	0.3041	0.0208
LN868538_g	0.0058	0.0110
Senegalimassilia	0.0065	0.0082
PAC001177_g	0.0095	0.0085
Fournierella	0.0001	
Monoglobus	0.0248	0.0043
PAC001286_g	0.0003	
Lachnospiraceae_uc	0.1090	0.0972
Pseudoglutamicibact	0.0025	
PAC001643_g	0.0005	

HQ452859_g	0.0006	0.0020
FN436095_g	0.0003	
Aggregatibacter	0.0002	0.0013
Raoultella	0.0133	0.0019
Paeniclostridium	0.0021	0.0009
Butyrivibrio_g1	0.0903	0.3257
EU471610_g	0.0001	
Victivallis	0.0128	0.0053
FJ681456_g	0.0003	
Bulleidia	0.0185	0.0069
Adlercreutzia	0.0010	0.0023
Lachnoanaerobaculu	0.0032	0.0002
PAC002510_g	0.0007	0.0003
Ruthenibacterium	0.1433	0.0469
PAC000395_g	0.0001	
Butyricimonas	0.0960	0.1461
Erysipelotrichaceae_	0.3307	0.1263
AM277970_g	0.0032	0.0008
Kocuria	0.0001	
Kandleria	0.0002	0.0002
PAC001241_g	0.0196	0.0005
Oxalobacter	0.0088	0.0059
Anaerotaenia	0.0001	0.0001
Agathobaculum	0.2818	0.3239
PAC001057_g	0.0040	0.0014
Campylobacter	0.0077	0.0036
Bergeyella	0.0002	
Coprococcus_g2	0.5176	0.4770
Granulicatella	0.0126	0.0071
PAC001472_g	0.0004	
AY858394_g	0.0001	
Holdemanella	0.0619	0.1894
PAC001166_g	0.0001	
Paraprevotella	0.0467	0.1158
Oribacterium	0.0059	0.0023
FJ269091_g	0.0004	0.0003
PAC001276_g	0.5211	0.6753
PAC002194_g	0.0004	0.0008
LLKB_g	0.1112	0.1574
Harryflintia	0.0017	0.0013
PAC001068_g	0.0319	0.0219
FTRU_g	0.0125	0.0278
Enorma	0.0005	0.0001
Eubacterium_g10	0.0002	
Coprobacter	0.0578	0.0436
PAC001699_g	0.0015	

Fusobacterium	0.0051	0.0086
FJ880790_g	0.0016	
PAC000744_g	0.0005	0.0003
Corynebacterium	0.0010	0.0002
Fructobacillus	0.0001	0.0004
AY442821_g	0.0042	0.0082
PAC000198_g	0.0039	
PAC001265_g	0.0229	0.0006
Janthinobacterium	0.0003	
Mesorhizobium	0.0001	
PAC002186_g	0.0002	0.0002
Citrobacter	0.0605	0.0016
Bifidobacterium	0.9024	1.18
PAC001687_g	0.0189	0.0201
PAC001144_g	0.0411	0.0227
Kosakonia	0.0007	0.0009
PAC001398_g	0.0342	0.0557
GU174072_g	0.0004	0.0020
Selenomonas	0.0005	
Sellimonas	0.0008	0.0020
PAC001231_g	0.0389	0.0030
Akkermansia	0.8477	0.7210
Coprobacillus	0.0027	0.0013
PAC002197_g	0.0078	0.0083
PAC000186_g	0.0366	
EU842424_g	0.0062	0.0043
PAC001134_g	0.0164	0.0155
Parasutterella	0.8028	0.8889
FJ848448_g	0.0019	
Comamonas	0.1439	0.9541
Syntrophococcus	0.0021	0.0031
EU622674_g	0.0001	
PAC001341_g	0.0008	
PAC000197_g	0.0024	0.0029
EU778925_g	0.0001	0.0004
Faecalicatena	0.0030	0.0012
LT821227_g	0.0063	0.0125
Eubacterium_g21	0.0230	0.0416
Veillonellaceae_uc	0.0002	0.0116
Eubacterium_g20	0.3585	0.1196
PAC001451_g	0.0179	0.0869
PAC001663_g	0.0539	0.0529
Prevotellaceae_uc	0.0730	0.0037
PAC001686_g	0.0046	0.0007
Eubacterium_g24	0.3217	0.4867
EU791177_g	0.0001	

Parvimonas	0.0020	0.0007
Lactonifactor	0.0003	
PAC001168_g	0.0055	0.0084
Eubacterium_g5	0.0228	0.0300
Eubacterium_g4	0.1201	0.0571
Barnesiellaceae_uc	0.0009	0.0005
Eubacterium_g8	0.4675	0.1679
Fusicatenibacter	0.1334	0.2827
Eubacterium_g17	0.1304	0.0486
Pyramidobacter	0.0028	
Peptostreptococcus	0.0003	0.0002
PAC002196_g	0.0056	0.0004
DQ673521_g	0.0001	
Klebsiella	0.1423	0.1187
PAC001651_g	0.0007	0.0058
Propionibacterium	0.0001	
AB264065_g	0.0011	
PAC001112_g	0.0003	0.0004
DQ456197_g	0.0020	0.0002
Fretibacterium	0.0003	
PAC001599_g	0.0002	0.0002
PAC000196_g	0.0115	0.0279
Mitsuokella	0.3618	0.1312
HQ806051_g	0.0021	
AB606290_g	0.0001	0.0007
PAC001100_g	0.0176	0.0225
PAC000742_g	0.0005	0.0006
PAC002199_g	0.0012	0.0015
Shuttleworthia	0.0005	
Sutterellaceae_uc	0.0011	0.0002
CCMM_g	0.0588	0.0648
Catonella	0.0394	0.0001
Franconibacter	0.0004	
Odoribacter	0.3066	0.4528
Pseudomonas	0.0002	0.0007
Staphylococcus	0.0035	0.0030
PAC002187_g	0.0001	0.0004
PAC000743_g	0.0018	0.0044
PAC000195_g	0.0628	0.0834
PAC001609_g	0.0417	0.0438
Microvirgula	0.0001	
Caproiciproducens	0.1721	0.1123
FJ881160_g	0.0002	0.0001
HM124264_g	0.0004	0.0012
PAC002360_g	0.0044	0.0048
PAC002152_g	0.0034	0.0020

AY212760_g	0.0045	
Emergencia	0.0023	0.0075
Dorea	0.4466	0.4043
Murimonas	0.0014	0.0024
AM420052_g	0.0002	0.0003
PAC001465_g		0.0005
JX095860_g		0.0002
HQ399867_g		0.0001
CYUO_g		0.0003
EU475449_g		0.0003
EU462118_g		0.0002
Salinimicrobium		0.0005
Hydrogenoanaerobac		0.0003
Coriobacteriaceae_uc		0.0005
PAC002181_g		0.0023
EU843993_g		0.0003
PAC002528_g		0.0001
JPZU_g		0.0001
PAC001500_g		0.0001
AX003092_g		0.0002
PAC001323_g		0.0029
Bacillus		0.0003
PAC001433_g		0.0006
Alloscardovia		0.0002
Victivallaceae_uc		0.0006
Mogibacterium		0.0002
Nesterenkonia		0.0002
Aminicella		0.0003
PAC001908_g		0.0004
Cosenzaea		0.0002
JRNA_g		0.0002
DQ238618_g		0.0002
AB506348_g		0.0001
AY854276_g		0.0009
Succinatimonas		0.0003
Gardnerella		0.0012
PAC001485_g		0.0012
EU474900_g		0.0002
PAC001108_g		0.0011
PAC001074_g		0.0005
PAC001266_g		0.0001
Vagococcus		0.0001
PAC001640_g		0.0018
Rhabdochlamydia		0.0002
NFJT_g		0.0017
Angelakisella		0.0002

HM124225_g	0.0006
Pseudopropionibacte	0.0001
Mobiluncus	0.0005
Micrococcus	0.0001
FJ507320_g	0.0693
PAC002141_g	0.0007
Clostridium_g21	0.0009
KE159571_g	0.0001
Catabacter	0.0002
Enterorhabdus	0.0004

Supplementary Table 5: Microbiome taxonomic composition at the Species level

	H. pylori -positive	*H. pylori* -negative
Bacteroides uniformis	3.57	4.83
Dialister PAC001040_s	1.35	
Parabacteroides merdae	1.06	1.02
Escherichia coli group	4.31	1.97
Bacteroides dorei	3.18	5.10
Megasphaera indica	1.01	
Prevotella_uc	3.86	2.44
Agathobacter rectalis	1.68	2.33
PAC001046_g PAC001046_s	1.12	1.62
Subdoligranulum PAC001173_s	1.14	1.16
Bacteroides vulgatus	6.67	4.64
Bacteroides massiliensis	1.07	
Prevotella PAC001304_s	5.52	6.60
Faecalibacterium prausnitzii group	5.44	6.37
Prevotella PAC001292_s	1.03	
Eubacterium_g23 PAC001051_s	1.05	
Dialister PAC001039_s	2.01	1.35
ETC(under 1% in average)	54.27	52.92
Eubacterium eligens group		1.47
Lactobacillus rogosae group		1.02
Succinivibrio FJ673381_s		1.98
Bacteroides stercoris		1.66
Dialister succinatiphilus		1.12

Supplementary Table 6: Taxonomic biomarker discovery- LEfSe analysis across H. pylori-pos and H. pylori-neg groups

Taxon name	Taxon rank	H. pylori pos	H. pylori neg	LDA effect size	P-value	P-value(FDR)
PAC001155_s (Ruminococcaceae)	Species	0.00000	0.27130	3.17	0.01873	0.045973
Blautia(g)	Genus	0.37517	0.59836	3.08	0.01395	0.047
PAC002309_g(Anaeroplasmataceae)	Genus	0.01335	0.18967	2.97	0.00625	0.0421875
PAC002309_s(Anaeroplasmataceae)	Species	0.01335	0.18967	2.97	0.00625	0.028125
Anaeroplasmatales	Order	0.01335	0.18967	2.95	0.00625	0.03375
AY985853_s (Bacteroidetes)	Species	0.01347	0.04797	2.38	0.01016	0.03918857
PAC000740_g (Lachnospiraceae)	Genus	0.03466	0.07499	2.37	0.04631	0.0480911
PAC000740_s(Lachnospiraceae)	Species	0.03466	0.07474	2.36	0.04631	0.04631
PAC001673_s (Desulfovibrio)	Species	0.00000	0.02066	2.04	0.04377	0.045453462
PAC001101_s(Ruminococcaceae)	Species	0.02358	0.00000	2.01586	0.04189	0.04712625
Howardella	Genus	0.02478	0.00221	2.044	0.02050	0.046125
PAC001265_s(Mollicutes)	Species	0.02363	0.00063	2.04909	0.03465	0.04455
PAC001265_o(Mollicutes)	Order	0.02363	0.00063	2.05388	0.03465	0.0467775
PAC001265_g(Mollicutes)	Genus	0.02363	0.00063	2.06144	0.03465	0.04923947
Blautia wexlerae	Species	0.10397	0.09961	2.31916	0.02430	0.0468642
Citrobacter	Genus	0.06255	0.00155	2.49401	0.01496	0.04708125
PAC001042_s(Prevotella)	Species	0.71674	0.10419	3.54156	0.02343	0.0486623

Supplementary Table 7: Comparative mean abundance of functional biomarkers across H. pylori-pos and H. pylori-neg groups

level 1 Pathways	*H. pylori*- neg	*H. pylori*- pos	Difference	P-value	FDR
Metabolism	0.436158392	0.454009084	0.01785	0.03127724	0.041702983
Environmental Information Processing	0.04326981	0.049418339	0.006148	0.04636963	0.046369627
Human Diseases	0.021897316	0.01599883	0.005898486	0.02728851	0.109154056
Cellular Processes	0.156010151	0.17399538	0.017985229	0.030309	0.060618

level 2 Pathways	*H. pylori*- neg	*H. pylori*- pos	Difference	P- value	FDR
Amino acid metabolism	0.436158392	0.454009084	0.01785	0.03127724	0.041702983
Signal transduction	0.04326981	0.049418339	0.006148	0.04636963	0.046369627
Infectious disease: viral	0.021897316	0.01599883	0.005898486	0.02728851	0.109154056
Cell growth and death	0.156010969	0.17399538	0.017985229	0.030309	0.060618

Pathway	level 3 Pathways	*H. pylori*- neg	*H. pylori*- pos	Difference	P- value	FDR
Ko00330	Arginine and proline metabolism	0.436158392	0.454009084	0.017850691	0.03127724	0.052128728
ko04016	MAPK signaling pathway	0.04326981	0.049418339	0.006148529	0.04636963	0.046369627
ko05164	Influenza A	0.021897316	0.01599883	0.005898486	0.02728851	0.068221285
ko04216	Ferroptosis	0.038642171	0.016198452	0.022444	0.01618254	0.080912685
ko04110	Cell cycle	0.117368798	0.157796928	0.04043	0.04443552	0.055544398

Supplementary Fig. 1 Count and rarefaction curves of the valid OTUs reads among all samples of both groups

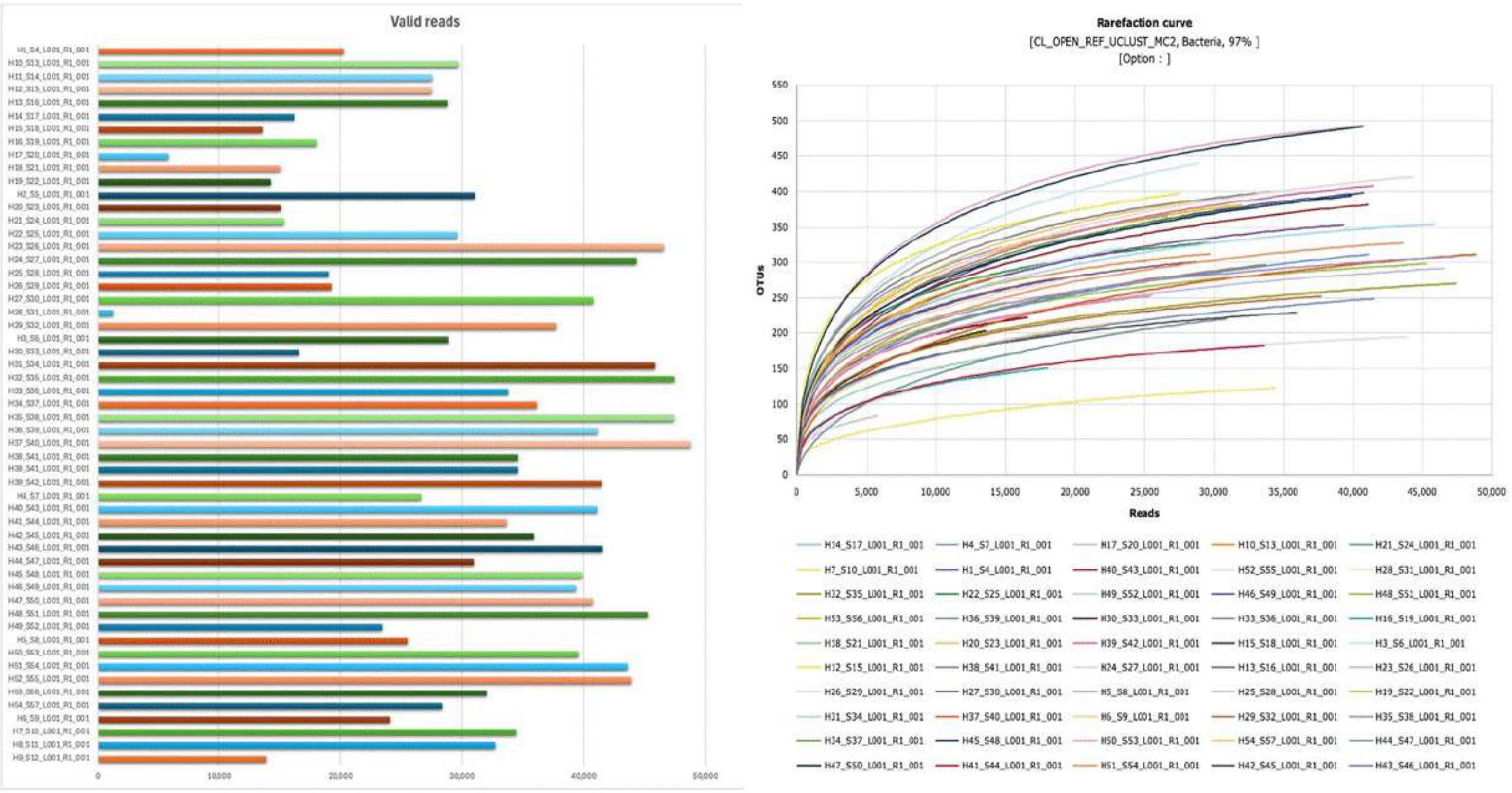

Supplemenatry Figure 2:Gut microbiota taxonomic composition among H. pylori-pos and -neg groups

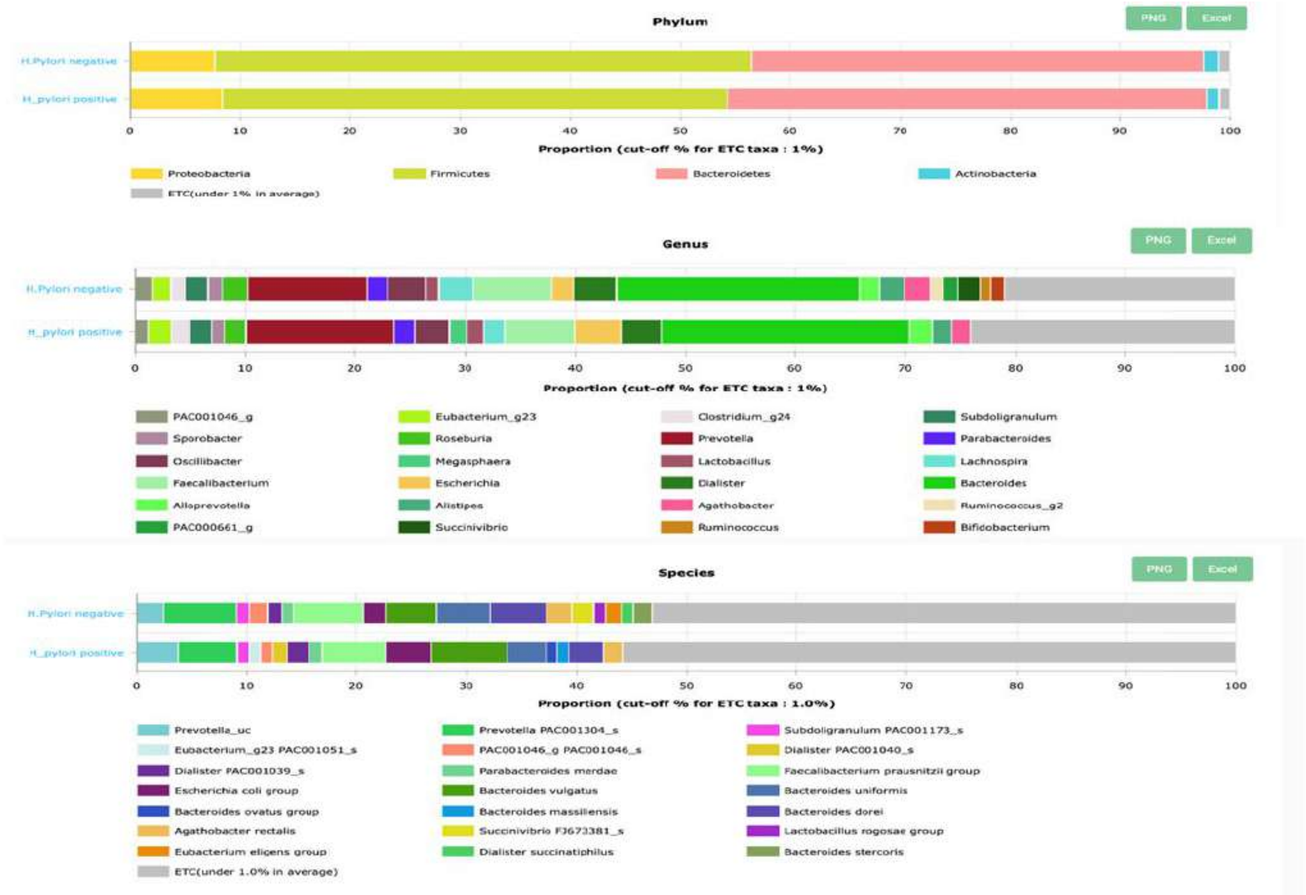

Supplementary Figure 3: Distribution of Level 1 KEGG functional pathways Cross H.pylori-pos and H. pylori-neg groups

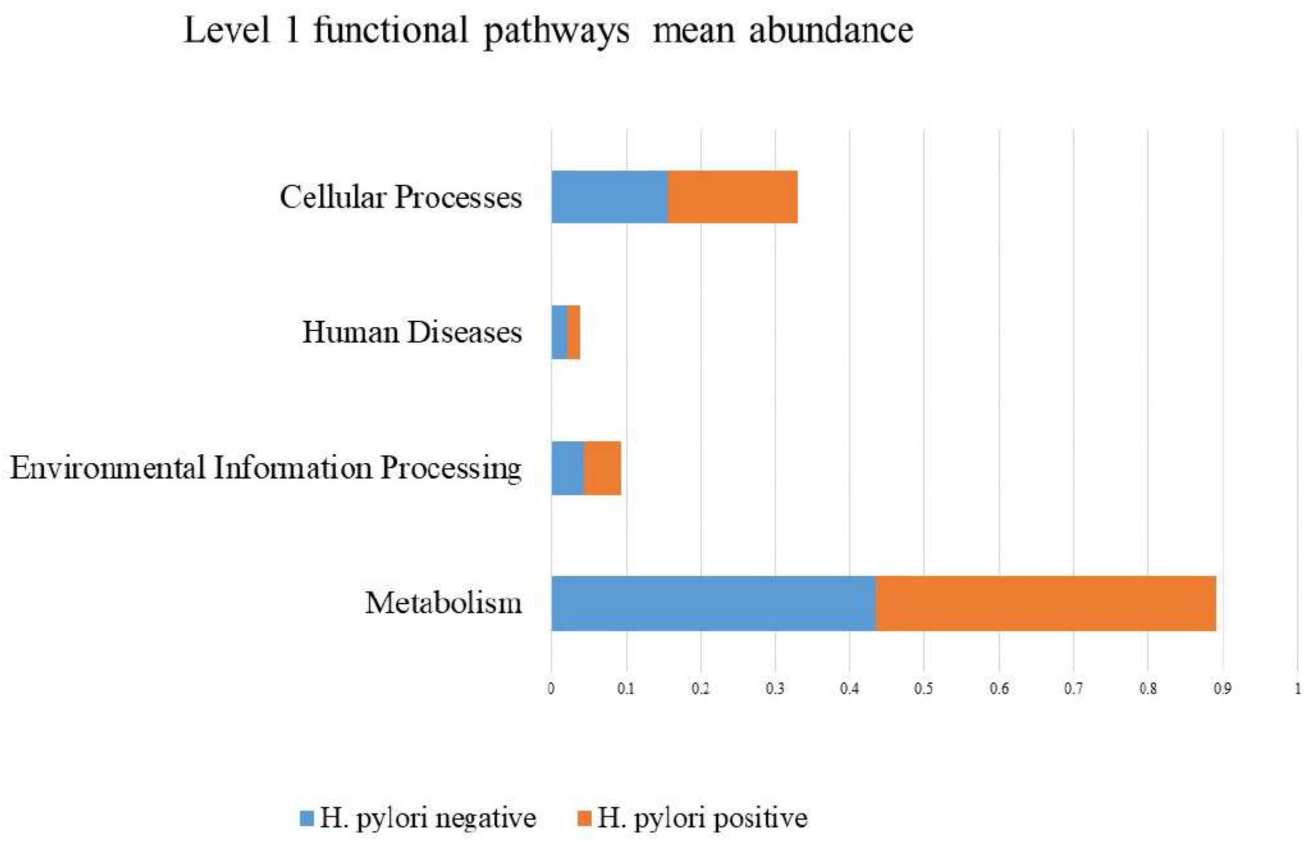

Supplementary Figure 4: Distribution of Gut microbiota functional pathways at level 2 KEGG databases across H. pylori-pos and -neg groups

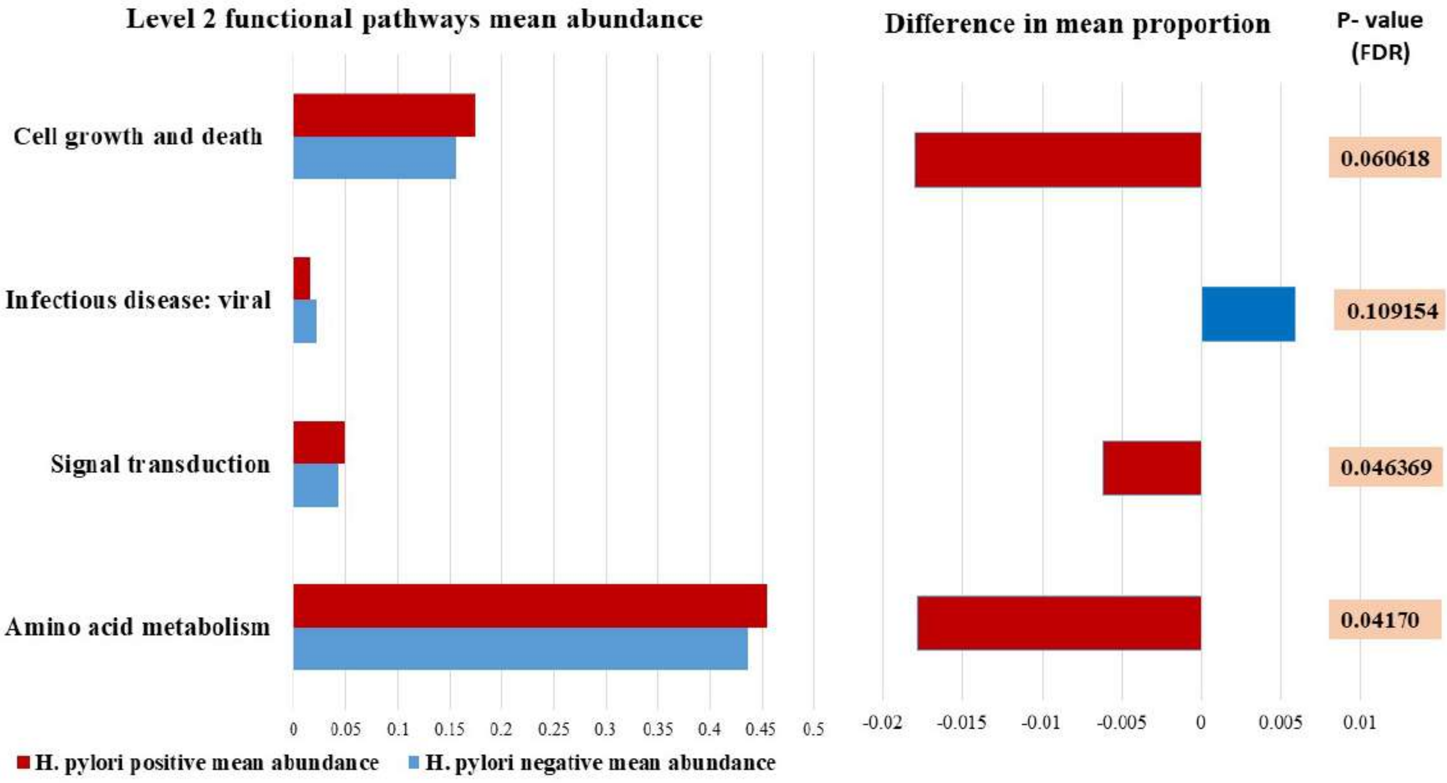

Supplement: Supplementary file 1 — Supplementary Material 1 [file 41598_2025_22788_MOESM1_ESM.pdf]
